# Responses of Caribbean Mangroves to Quaternary Climatic, Eustatic, and Anthropogenic Drivers of Ecological Change: A Review †

**DOI:** 10.3390/plants11243502

**Published:** 2022-12-13

**Authors:** Valentí Rull

**Affiliations:** Botanic Institute of Barcelona (IBB), Spanish National Research Council (CSIC), Pg. del Migdia s/n, 08038 Barcelona, Spain; vrull@csic.es

**Keywords:** mangroves, Caribbean, climate change, sea levels, human disturbance, palynology, biotic responses, Pleistocene, Holocene

## Abstract

Mangroves are among the world’s most threatened ecosystems. Understanding how these ecosystems responded to past natural and anthropogenic drivers of ecological change is essential not only for understanding how extant mangroves have been shaped but also for informing their conservation. This paper reviews the available paleoecological evidence for Pleistocene and Holocene responses of Caribbean mangroves to climatic, eustatic, and anthropogenic drivers. The first records date from the Last Interglacial, when global average temperatures and sea levels were slightly higher than present and mangroves grew in locations and conditions similar to today. During the Last Glaciation, temperatures and sea levels were significantly lower, and Caribbean mangroves grew far from their present locations on presently submerged sites. The current mangrove configuration was progressively attained after Early Holocene warming and sea level rise in the absence of anthropogenic pressure. Human influence began to be important in the Mid-Late Holocene, especially during the Archaic and Ceramic cultural periods, when sea levels were close to their present position and climatic and human drivers were the most influential factors. During the last millennium, the most relevant drivers of ecological change have been the episodic droughts linked to the Little Ice Age and the historical developments of the last centuries.

## 1. Introduction

**Figure 1:**
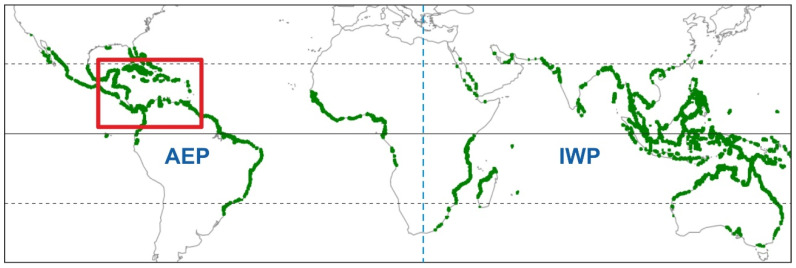
World map of mangroves (green fringes) indicating the Caribbean region (red box). The African continental barrier between the Atlantic-Eastern Pacific (AEP) and Indo-West Pacific (IWP) regions is represented by a dotted blue line in the center. Base map from Ref. [1].

Mangroves are transitional land-sea forested wetland ecosystems distributed across the whole tropical/subtropical zone between approximately 25° N and 25° S (Figure 1). These ecosystems are especially adapted to intertidal conditions and are found in more than 120 countries on all continents, with a global extent of over 140,000 km^2^, which represents approximately 75% of tropical coastlines. Asia is the continent with the highest proportion of mangroves (~38%), followed by Latin America and the Caribbean (~20%), Africa (~20%), Oceania (~12%), North America (~8%) and European/USA Overseas Territories (<1%) [1,2,3,4]. Mangroves have been considered keystone ecosystems, as they provide habitat for many terrestrial and marine species (algae, mollusks, sponges, crustaceans, fishes, mammals, birds, reptiles, amphibians), support neighboring ecosystems (coral reefs, seagrasses), and are globally linked via atmospheric processes, participating in the global carbon cycle as an important carbon sink [5,6].

Currently, mangroves are among the world’s most threatened ecosystems [7]. Since the 1980s, these forests have experienced a global cover loss by deforestation of 25–30%, which would lead to their disappearance within the next century [8,9]. Major threats are shrimp and fish aquaculture, firewood and charcoal extraction, agriculture, urbanization, and global-change-driven effects such as increased atmospheric CO_2_ concentration, sea level rise, global warming, changing precipitation patterns, increasing extreme weather events, invasion by alien species, and biodiversity depletion, among others [10,11,12,13]. Understanding how mangrove ecosystems respond to natural and anthropogenic drivers of change is essential for informing conservation and restoration practices. In this sense, the responses of mangroves to climatic, eustatic, and anthropogenic pressures of the last centuries and millennia may be of great utility, as they provide a natural laboratory to study the potential effects of the main threats [14,15,16].

Globally, mangroves are subdivided into two main biogeographical units, namely the Atlantic-East Pacific (AEP) and the Indo-West Pacific (IWP) regions, with contrasting biodiversity patterns, separated by the African continental barrier (Figure 1). The IWP is the richest region by far. Of the 69 known mangrove plant species (excluding hybrids), 54 (17 families, 24 genera) live in the IWP region, and 17 (9 families, 11 genera) occur in the AEP region [17]. Especially relevant are the so-called mangrove-forming tree species, which dominate the forest canopy and are primarily responsible for the physical structure of mangrove formations. The most important mangrove-forming tree species are from the genera *Rhizophora* (Rhizophoraceae) and *Avicennia* (Acanthaceae), which are globally distributed, the first with 6 species (2 in the AEP region and 4 in the IWP region) and the second with 8 species (3 in the AEP region and 5 in the IWP region) [18].

**Figure 2:**
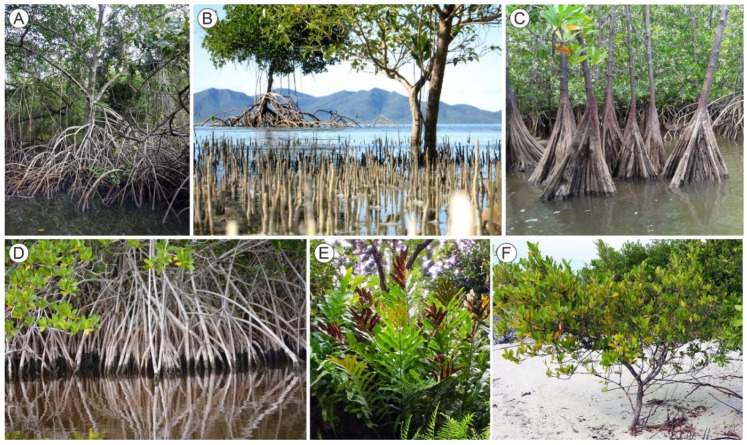
Examples of mangrove-forming trees from the Caribbean region. (**A**) *Rhizophora mangle*; (**B**) *Avicennia germinans*; (**C**) *Pelliciera rhizophorae*; (**D**) *Laguncularia racemosa*; (**E**) *Acrostichum aureum*; (**F**) *Conocarpus erectus*. Modified from Ref. [20].

The Caribbean mangroves belong to the AEP region (Figure 1), where *Rhizophora* is represented by *R*. *mangle* and *R*. *racemosa*, whereas *Avicennia* is represented by *A*. *bicolor*, *A*. *germinans* and *A*. *schaueriana*. Other Caribbean mangrove-forming trees are *Laguncularia racemosa, Conocarpus erectus* (Combretaceae), and *Pelliciera rhizophorae* (Tetrameristaceae), which are exclusive to the AEP region [18]. The Caribbean mangrove species exhibit a characteristic zonation following a land-sea gradient created mainly by tidal influence and salinity. Mangrove forests with *Rhizophora*, *Avicennia*, *Laguncularia,* and *Pelliciera* grow in the intertidal zone permanently flooded by saline waters, where they develop a complex network of aerial and submerged habitats for a diversity of organism’s characteristic of the mangrove ecosystem (Figure 2). In more inland brackish-water environments under the influence of spring tides, the back-mangrove swamp communities are usually characterized by *C*. *erectus*, the fern *Acrostichum aureum,* and aquatic herbs. The alluvial plains flooded by freshwaters are dominated by *Mauritia flexuosa* and other palms, which mark the transition to continental non-flooded environments covered by a diversity of tropical forests and savannas [19].

The Caribbean was the cradle of Neotropical mangroves, which originated in the Eocene and were dominated by the ancestor of the extant *P*. *rhizophorae* [20]. The global environmental disruption that occurred in the Eocene/Oligocene transition (EOT) was likely involved in the abrupt replacement of the primeval autochthonous *Pelliciera* mangroves by the alien *Rhizophora* mangroves, which originated in the IWP region and arrived by long-distance dispersal through the Atlantic Ocean [21]. Since then, *Rhizophora* mangroves have dominated the Neotropical coasts with no significant evolutionary changes, whereas *Pelliciera* has been progressively restricted to its present relict area around the Central-American Panama Isthmus [22]. Present-day *Rhizophora* mangroves are the result of a progressive Neogene (Miocene, Pliocene) and Quaternary diversification of the Oligocene mangroves with no apparent extinctions [23].

The main trends in the Paleogene (Eocene-Oligocene) and Neogene (Mio-Pliocene) evolution of Caribbean mangroves and the potential environmental drivers involved are relatively well known. However, a comprehensive study of the ecological and evolutionary developments that occurred in the Quaternary (Pleistocene–Holocene) is still lacking, which hinders understanding how Quaternary natural and anthropogenic drivers have contributed to the shaping of extant Caribbean mangrove ecosystems. This paper reviews the available Pleistocene and Holocene paleoecological records to fill his gap, with emphasis on the following aspects: (i) the influence of Pleistocene climatic cycles and the associated eustatic shifts; (ii) the role of postglacial warming and sea-level rise, which occurred roughly synchronously with human colonization of the American continent; (iii) the effect of Holocene climatic shifts, with emphasis on the hydrological balance (precipitation/evaporation ratio); (iv) the potential consequences of prehistoric human activities; (v) the influence of European contact and further occupation; and (vi) the occurrence of eventual climatic-anthropogenic feedbacks and synergies, especially during the last millennium.

The review begins with a description of the study area (Section 2), with emphasis on the main environmental features, followed by a characterization of extant mangroves in biogeographical, floristic, and ecological terms. The next Section 3 is an account of the main Pleistocene and Holocene climatic and eustatic changes recorded in the Caribbean area, followed by a summary of the patterns of human settlement, aimed at providing the suitable context for analyzing the response of mangrove communities to these natural and anthropogenic drivers. The core of the paper is Section 4, where these responses are reviewed and analyzed using a chronological approach. This section begins with a summary of the available studies on modern analogs, which are needed for a reliable interpretation of past records. Then the database used in this study, which has been compiled from the available literature and includes almost 60 Late Pleistocene and Holocene mangrove records across the Caribbean coasts, is introduced and described. The mangrove response analysis is conducted using this database, following a chronological sequence: Late Pleistocene, Last Glaciation, Middle and Late Holocene (Early Holocene records are lacking), and last millennium. Finally, Section 5 summarizes the main findings of this review and proposes some prospects for future studies.

## 2. Study Area

**Figure 3:**
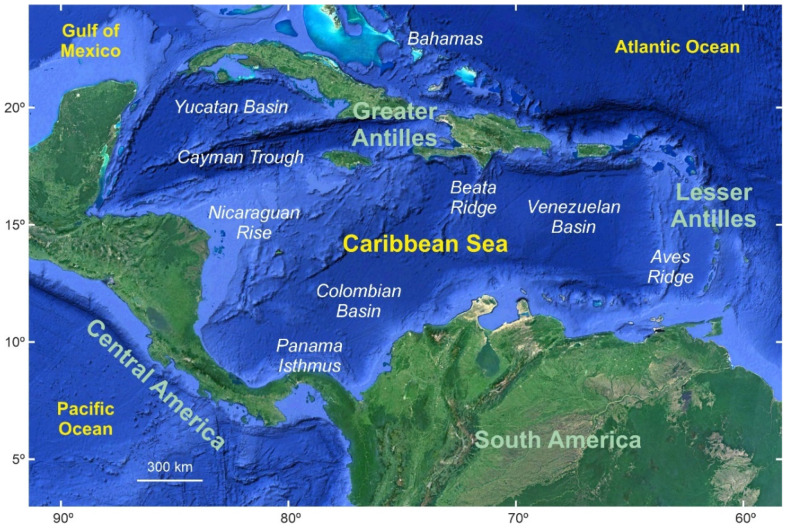
A Google Earth map showing the Caribbean region *sensu lato* considered in this study and the main physiographic features.

The area studied here can be referred to as the Caribbean region *sensu lato* or the circum-Caribbean region and includes the Atlantic and Pacific coasts of the Antilles, Central America, and northern South America situated between approximately 5°–25° N and 60°–90° W (Figure 3). Politically, this region covers over 20 countries, including some European and North American overseas possessions (Table 1).

### 2.1. Climate and Sea Level

#### 2.1.1. Temperature and Precipitation

**Figure 4:**
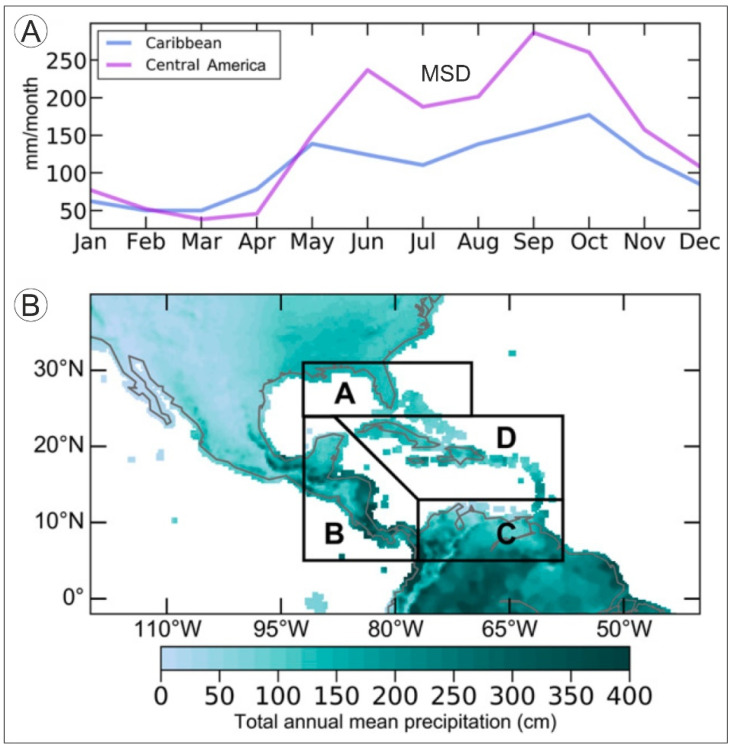
Caribbean precipitation patterns. (**A**) Seasonal variations in Central America and the Caribbean islands. (**B**) Total annual precipitation patterns across the study area (A, Florida region; B, Central America; C, northern South America; D, Caribbean islands). MSD, mid-summer drought. Modified from Ref. [26].

As usual in tropical climates, annual variations of average temperatures are small, ranging from 2 °C to 7 °C, whereas rainfall shows high spatiotemporal variability [24]. Annual average air temperatures range between approximately 22 °C and 28 °C, with minima in December and January and maxima between June and September. Sea Surface Temperatures (SSTs) are between 25 °C (January–December) and 30 °C (July) [25]. Total annual precipitation is highly variable, ranging from <1000 to >4000 mm/year (Figure 4), with minimum values in the Lesser Antilles and the northern South American coasts and maxima in Central America and NW South America [26], where one of the most humid areas of the world—the Chocó region, which may attain values of 13,000 mm/year [27]—lies. A clear and consistent warming trend of ~0.1 °C/decade, on average, has been observed since 1900, which is consistent with the global warming of the past century [28,29]. In contrast, no significant increasing or decreasing rainfall trends have been documented during the same period, in which precipitation patterns have been strongly dominated by interannual and decadal variability [25,28].

The most characteristic feature of Caribbean climatology is the annual precipitation regime, which shows a typical bimodal pattern characterized by two maxima (~150–250 mm/month) in May–June and September–October, and a minimum (~50 mm/month) in December–April (Figure 4). During the wet season (May–October), a relatively dry period occurs between July and August known as the Mid-Summer Drought (MSD) [26,30]. This strong seasonality is due to the interplay of a number of larger-scale climatic features, notably the North Atlantic Subtropical High (NASH)—also known as the Azores High (AH)—the eastern Pacific Intertropical Convergence Zone (ITCZ), the Atlantic Warm Pool (AWP), and the Caribbean Low-Level Jet (CLLJ). The NASH and the ITCZ are the most important moisture facilitators, and their seasonal migrations, expansions and contractions are the main reasons for the bimodal precipitation pattern. The first affects primarily the central and eastern Caribbean islands (zone C of Figure 4), whereas the second is more influential in the western sector (zone B of Figure 4). The AWP and CLLJ modulate the extent of moisture provided by the NASH and ITCZ seasonal dynamics [31]. The causes of the MSD are still poorly understood.

#### 2.1.2. Meteorological Hazards

The Caribbean region is prone to climatically-driven natural disasters causing significant economic damages. Tropical cyclones (TCs), or hurricanes, and the associated extreme rain events, floods, and landslides constitute a major threat [32,33]. The hurricane season spans June to November, when the North Atlantic easterly trade winds are weaker and SSTs are above 26 °C, which create the ideal conditions for cyclonic activity [25]. A significant increase in hurricane activity and in the amount of intense storms has been observed since 1995 [34]. The El Niño-Southern Oscillation (ENSO) plays an important role in modifying hurricane activity by increasing (La Niña conditions) or decreasing (El Niño conditions) the number and intensity of these events.

The Caribbean region, especially the islands, is also affected by short but intense droughts that cause important economic losses affecting agriculture (the main basis of the regional economy), water availability, hydroelectric power generation, and tourism [35,36]. Drought occurrence and intensity show significant spatiotemporal heterogeneity that prevents the finding of regularities useful for prediction. For example, different Caribbean areas have different periods of drought occurrence, ranging from 2–3 to 9–13 years. In spite of this, correlations with tropical and equatorial Pacific SSTs suggest a potential link with El Niño events. It has not been possible to clearly define long-term trends of variation, as some Caribbean areas show drying trends while others show a tendency toward more wet climates [25].

In general, of the 370 meteorological disasters reported for the period 1960–2013 in 22 Caribbean countries, TCs have been the most frequent (~72%) followed by riverine flooding (~16%). During the same period, TCs accounted for 95% of the economic losses. It is also worth mentioning that the occurrence of natural disasters associated with meteorology has significantly increased from <2 to >10 events per year before and after 1980 [32].

#### 2.1.3. Sea Level

It has been estimated that global sea-level rise rates have accelerated from 1.7 mm/year during the 20th century to 3.2 mm/year since the beginning of the present century [37]. In the Caribbean Sea, rising rates of 1.8 mm/year and 2.5 mm/year have been measured for the periods 1950–2009 and 1993–2010, respectively [38,39]. However, there are significant departures from these figures, showing high spatial heterogeneity. The extremes are 0.3 mm/year on the northern South American coasts of Venezuela and 10.8 mm/year in Haiti (Hispaniola). These local differences have been attributed to post-glacial rebound and local tectonics [38,39].

#### 2.1.4. Future Projections

Current global change estimates for the Caribbean region predict a warming of 1–3.5 °C by the end of this century in both land and sea environments, with the highest warming rates in terrestrial settings, along with an increased frequency of temperature extremes. According to the same modeling approaches, a moderate drying trend consisting of a 20–30% reduction in rainfall will likely occur, accompanied by increases in heavy rainfall events and in the number of consecutive hot and dry days [40,41,42]. It is estimated that storms and hurricanes will also increase in frequency and intensity, as predicted by global projections. Regarding sea levels, Caribbean forecasts are also in agreement with global estimates of a 0.4–0.6 m increase relative to 1986–2005, although a few studies suggest that increases above 1 m should not be disregarded [43,44].

In addition to a multitude of human affairs, these events may impact terrestrial and marine ecosystems, and biodiversity. For example, increasing temperatures may foster coral bleaching, promote blooms of invasive species, or favor the northern migration of Caribbean fish and coral reefs. Decreasing rainfall may affect patterns of seed production, germination survival and development, thus increasing seedling mortality. Increased flood, storm and hurricane frequency may enhance the degradation of coastal wetlands and forests (notably mangroves), and reduce their natural filtering and buffering capacity. This may enhance habitat loss and contribute to the degradation of other ecosystems, such as coral reefs, ultimately leading to biodiversity depletion. Sea-level rise may also affect mangroves by promoting their landward migration and enhancing coastal erosion [25].

### 2.2. Mangrove Communities

#### 2.2.1. Geographical Extent

**Figure 5:**
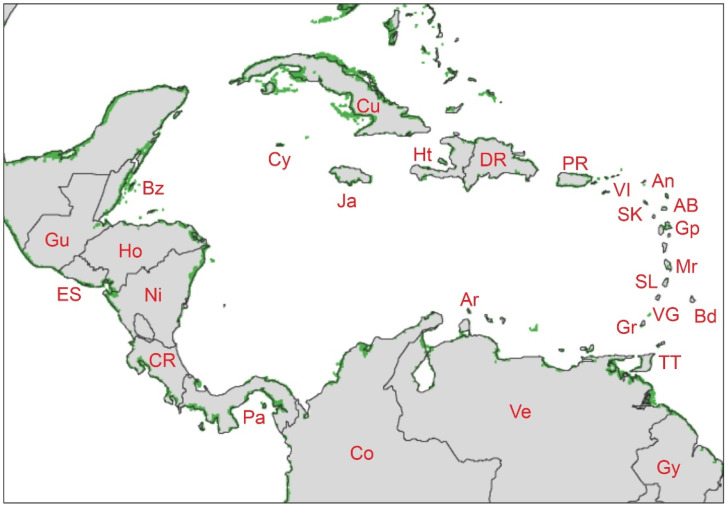
NASA Landsat 5-TM image of the Caribbean mangrove areas (green patches) using the data of Ref. [4]. Abbreviations from Table 1. Base map downloaded from https://earthobservatory.nasa.gov/images/47427/mapping-mangroves-by-satellite (last accessed 3 October 2022).

Current estimates on the total extent of mangroves in the study area are between 14,000 and 15,000 km^2^, which represents 10–11% of the total world’s mangrove extent (Table 1, Figure 5). The countries with more extensive mangroves are Cuba and Venezuela (over 3000 km^2^, according to the latest estimates), followed by Colombia and Panama (1500–2600 km^2^); all other countries are below 700 km^2^ and most of them do not reach 100 km^2^ of mangrove cover. The above-quoted significant reduction in the total world’s mangrove cover documented for the last decades has also affected the Caribbean region, where mangrove extent has been reduced by ~34% (~21,000 to ~14,000 km^2^) in three decades (1980–2010) (Table 1), at an average rate of 1.14% (~240 km^2^) per year. These estimates exceed by almost 10% the world’s average (~25%) and have progressed at rates 1.4 faster than global ones (0.85%). In the Neotropics, the most relevant drivers of this reduction in mangrove cover have been urbanization, damming, agriculture, forestry, tourism, fisheries, salt production and shrimp farming [45].

**Table 1:** Mangrove extent (km^2^) by country/island in the Caribbean region, comparing the most updated estimates (2010) with those of 1980. Mexico has not been included because most mangroves in this country are outside the Caribbean region, and estimates only for its Caribbean coasts are unavailable.

Code	Country/Island	1980 [46]	2010 [4]
An	Anguilla (UK)	0.90	1.00
AB	Antigua and Barbuda	15.70	8.63
Ar	Aruba	4.20	0.26
Bd	Barbados	0.30	0.14
Bz	Belize	785.00	445.07
Cy	Cayman Islands (UK)	10.10	41.48
Co	Colombia	4400.00	2622.12
CR	Costa Rica	634.00	364.75
Cu	Cuba	5374.00	3328.16
DR	Dominican Republic	344.00	187.41
ES	El Salvador	467.00	375.89
Gr	Grenada	2.95	1.90
Gp	Guadeloupe (France)	30.00	37.13
Gu	Guatemala	186.00	235.23
Gy	Guyana	910.00	286.40
Ht	Haiti	178.00	144.32
Ho	Honduras	1525.00	597.32
Ja	Jamaica	120.00	94.11
Mr	Martinique (France)	19.00	20.52
Ni	Nicaragua	1034.00	739.88
Pa	Panama	2500.00	1533.37
PR	Puerto Rico	76.50	86.85
SK	Saint Kitts and Nevis	0.85	0.28
SL	Saint Lucia	2.00	16.40
VG	Saint Vincent and the Grenadines	0.55	0.31
TT	Trinidad and Tobago	75.00	76.96
Ve	Venezuela	2600.00	2753.25
VI	Virgin Islands (US and UK)	10.10	20.53
	**Caribbean Total**	**21,295.05**	**13,999.14**
	**World total**	**187,940.00**	**140,258.86**
	**Caribbean/World (%)**	**11.33**	**9.98**

#### 2.2.2. Floristics

**Figure 6:**
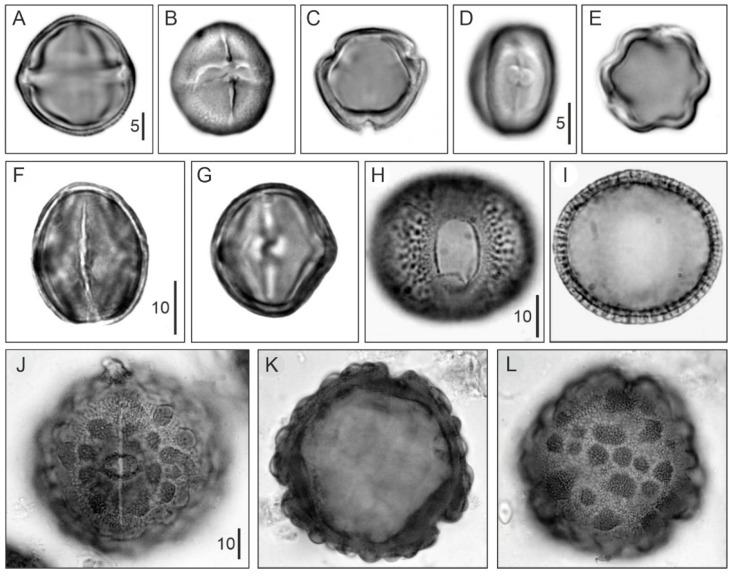
Pollen morphology of the main components of Caribbean mangroves. (**A**–**C**) *Rhizophora mangle* (red mangrove); (**D**,**E**) *Conocarpus erectus* (buttowood); (**F**,**G**) *Laguncularia racemosa* (white mangrove); (**H**,**I**) *Avicennia germinans* (black mangrove); (**J**–**L**) *Pelliciera rhizophorae* (tea mangrove). Measurements (vertical lines) in μm. Modified from Ref. [20].

Floristically, the Neotropical mangrove formations have been subdivided into 30 phytosociological associations clustered in five groups/subgroups [47]. These associations are dominated/sub dominated by species of the previously mentioned mangrove-forming trees of the genera *Rhizophora*, *Avicennia*, *Laguncularia* and *Conocarpus*. In addition, 85 species have been found that are exclusive to either one or another group and can be considered diagnostic species in this sense (Table 2). Group A encompasses associations where the main mangrove-forming trees are *A*. *germinans* (black mangrove) and *L*. *racemosa* (white mangrove), whereas *R*. *mangle* (red mangrove) is poorly represented. Associations from group B are dominated by either *C*. *erectus* (buttonwood) (B_1_) or *A*. *germinans* (B_2_). The most important tree in group C is *R*. *mangle*, either alone (C_1_) or in combination with *L*. *racemosa* and, in a single case, with *P*. *rhizophorae* (tea mangrove) (C_2_). As this paper uses mainly palynology to reconstruct the Quaternary history of Caribbean mangroves and their responses to environmental changes, pollen images of these species are provided in Figure 6.

#### 2.2.3. Biogeography

**Figure 7:**
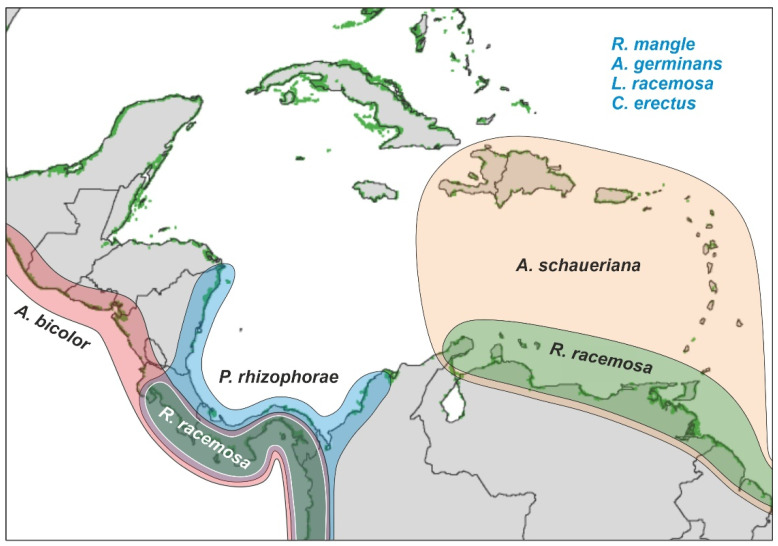
Approximate geographical range of the most important Neotropical mangrove-forming trees in the Caribbean region. Species distributed across the whole study area are indicated in blue in the upper right. *A*, *Avicennia*; *C*, *Conocarpus*; *L*, *Laguncularia*; *R*, *Rhizophora*. Composed from Refs. [49,50].

The most important Caribbean mangrove species of the above associations (*R*. *mangle*, *A*. *germinans*, *L*. *racemosa,* and *C*. *erectus*) are widespread across the whole region considered in this study. Other *Rhizophora* and *Avicennia* species, such as *R*. *racemosa*, *A*. *bicolor* and *A*. *schaueriana*, have more restricted distributions (Figure 7). The case of *P*. *rhizophorae* is especially noteworthy, as its present distribution area is considered a relic of the former range of *Pelliciera*, which was widespread across the whole Neotropics during the Miocene. The potential causes for this significant reduction have long been discussed and include low tolerance to environmental stressors (light, temperature, salinity, sea-level shifts) and competition with *Rhizophora* [23,48]. It has also been proposed that this relictualization could be considered the final stage of a long-lasting taxon cycle and, hence, the prelude to the extinction of the species [22].

#### 2.2.4. Ecology

Overall, *R*. *mangle* and the communities it develops are dominant in the Caribbean region. A thorough review of this species has recently been published [51], from which the following overview has been derived. *R*. *mangle* is typical of the intertidal seaward mangrove fringe but can also be found at the mouths of estuaries, on tidal creek banks and in the margins of upper reaches. The preferred substrate is soft mud or fine silt-clay from low-energy coastlines free of strong waves and high tidal amplitudes and not submitted to intense erosion. In contrast to the relative stability of the substrate, physiological stress is common in mangrove habitats, as manifested in variable salinity and nutrient availability, high temperatures, light intensity and anoxic sediments. *R*. *mangle* has developed a variety of physiological strategies to adapt to these extreme and changing conditions.

The red mangrove can tolerate a wide range of temperatures and is limited mainly by sustained freezing conditions, which allow this species to live from the Equator to the 16 °C latitudinal isotherm. Regarding salinity, although *R*. *mangle* typically occurs in values around seawater (~35‰), it is able to develop on aquatic environments ranging from freshwaters (0‰) to hypersaline waters (90‰). Nitrogen and phosphorus are the main nutrients limiting the growth of the red mangrove. Deficits in these macronutrients, especially phosphorous, may lead to the development of dwarf mangrove forests. Mangrove soils are usually anoxic and rich in sulfides, due to the active oxidation of the high amounts of sedimentary organic matter carried by rivers. *R*. *mangle* has adapted to these conditions by maintaining a high biomass of aerial roots (pneumatophores) able to perform direct gas exchange with the atmosphere (Figure 2). These roots also act as sediment traps and contribute to coastal accretion, which may be especially important in a sea level rising scenario like the present.

**Figure 8:**
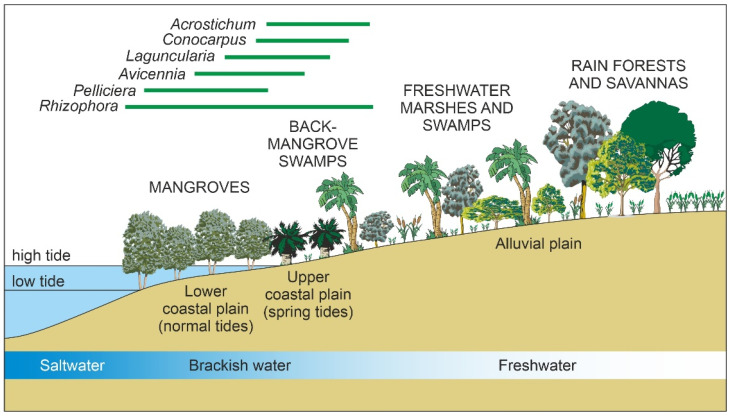
Idealized transect showing the typical coastal zonation of Caribbean mangroves. The approximate ranges of the most important mangrove elements are indicated by green lines. Modified from Ref. [20].

Due to the harsh environmental conditions, mangrove communities organized around *R*. *mangle* and associated tree species are not particularly biodiverse, and the understory below the forest canopy is poorly developed, consisting of a few herbs, shrubs, and vines. A characteristic land-sea community zonation develops because of the interplay among a diversity of biotic and abiotic factors. Among abiotic drivers, topography, tidal influence and the resulting salinity gradient have been especially highlighted. Competition among mangrove species and seed dispersal are the main biotic factors invoked to explain mangrove zonation [52,53,54,55,56]. The most common mangrove zonation pattern, from the outer fringe landward, is *R. mangle*—*A. germinans*—*L. racemosa*—*C. erectus*, overlapping back-mangrove brackish-water swamp communities and followed by freshwater marsh communities and inland rainforests and savannas (Figure 8). When *P*. *rhizophorae* is present, it thrives close to the outer fringe, within the *Rhizophora* canopy, which provides protection against extreme light and salinity conditions [57]. The zonation pattern may be subjected to small-scale variations due to local heterogeneities in the influencing factors. In some cases, only slight variations such as shifting positions in *A*. *germinans* and *L*. *racemosa* along the gradient occur [58,59]. In other cases, low environmental variability and the absence of permanent freshwater sources may lead to the lack of any zonation pattern at all and a nearly homogeneous mangrove composition [60].

*A*. *germinans* can live in fresh or hypersaline waters and has different pneumatophores [58] (Figure 2). This species is a mangrove pioneer in the upper intertidal fringe and is able to colonize unstable mud banks, where it develops temporary monospecific communities without understory vegetation [61]. However, propagules cannot establish on either permanently flooded or on dry soils and the optimal development of seedlings occurs at sites where the water level is near the surface and salinity is not high [58]. *L*. *racemosa* rarely dominates mangrove stands and occupies a narrow ecological niche on nutrient-rich elevated soils where tidal flooding is moderate to infrequent, and freshwater inputs are common, under intermediate salinity conditions (15–20‰) [59]. Beyond these limits, the species is unable to compete with other mangrove trees, notably *Rhizophora*. Occasional dominance of *L*. *racemosa* in some mangrove communities has been attributed to high concentrations of nutrients, especially phosphorus and nitrogen [62]. The white mangrove is less cold tolerant than *Rhizophora* and *Avicennia*, which limits its latitudinal range. *C*. *erectus* occurs on elevated terrain, in the ecotone between mangroves and upland tropical forests, under salinities not exceeding 10%. This species does not develop pneumatophores, is unable to live on flooded soils, and is noteworthy for its resistance to high temperatures [63].

## 3. The Quaternary

The response of Caribbean mangroves to Quaternary climatic shifts is a major target of this paper, and therefore, an overview of the main climatic shifts occurred during this geological period is essential. First, it is important to emphasize that when the Quaternary started, 2.6 million years ago (Ma), the Panama Isthmus was already closed and the circulation between the Caribbean Sea and the Pacific Ocean was interrupted. A debate exists about the exact chronology of the isthmus closure, but all reconstructions agree that the Pacific seaway was already closed by 2.8 Ma [64], before the onset of Quaternary climatic changes. Therefore, Atlantic circulation has been a major driver of Caribbean climatic shifts since then. From a biogeographical perspective, the formation of the Panama Isthmus implied the creation of a novel pathway for terrestrial dispersal across the American continent and an unprecedented barrier for marine organisms, which provided a very different paleogeographic ecological and evolutionary framework [65]. For mangroves, this new scenario would have provided new opportunities for both variance across the newly created Central American barrier [66,67,68,69,70] and gene flow along the Caribbean and Atlantic coastal currents, which are the most important dispersal agents for mangroves [71]. This section focuses on vegetation-independent evidence for climatic and eustatic changes, to avoid circularity when analyzing the responses of mangroves to these environmental drivers.

### 3.1. Pleistocene Climatic and Sea-Level Changes

**Figure 9:**
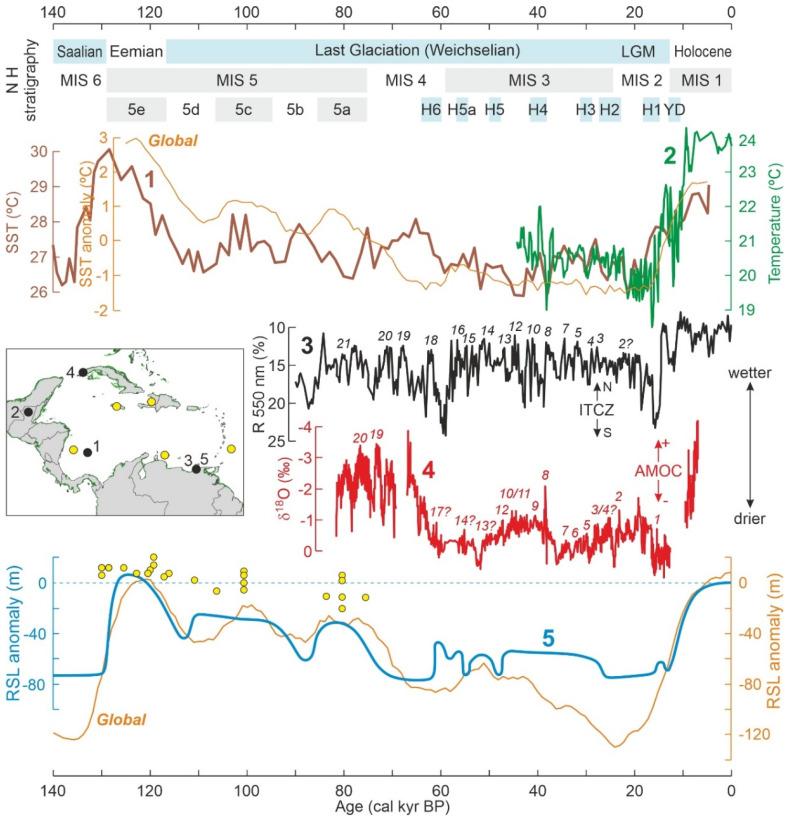
Temperature, moisture and sea level changes during the last glacial cycle in the Caribbean region, compared with global reconstructions. (1) Sea surface temperature (SST) reconstruction from core ODP-999A (Colombian Basin) [79]; (2) Land temperature reconstruction from Lake Petén Itzá (Guatemala) [80]; (3) Millennial ITCZ migration as recorded in the reflectance (R) measures of core ODP-1002 (Cariaco Basin, Venezuela) [81], numbers are the Dansgaard-Oeschger (D-O) events [82]; (4) Oxygen isotope ratio of speleothem Cuba Medio from the Santo Tomás cave (Cuba) [83], numbers are D-O events [82]; (5) Relative sea level (RSL) reconstruction based on seismic stratigraphy from the Venezuelan Gulf of Cariaco (blue line) [84] and several coral terraces from Barbados, Bonaire, Cayman, Curaçao, Haiti and San Andrés (Colombia) (yellow dots) [85,86,87,88,89]. Global temperature and sea-level reconstructions from Refs. [90,91], respectively. Northern Hemisphere (NH) glacial stratigraphy based on Refs. [92,93,94]. AMOC, Atlantic Meridional Overturning Circulation; H1-H6, Heinrich events; ITCZ, Intertropical Convergence Zone; LGM, Last Glacial Maximum; MIS, Marine Isotope Stages; YD, Younger Dryas.

Pleistocene glacial-interglacial cycles affected not only the temperate and polar zones but also the tropics [72] and, hence, the Caribbean region, which was subject to significant temperature shifts, moisture reorganizations and sea-level oscillations during this geological period [73,74,75,76]. The oldest Pleistocene Caribbean mangrove records found in the literature review performed for this paper date from ~130 kyr BP [77], which roughly coincides with the onset of the last glacial cycle, from the Last Interglacial—the Eemian or Marine Isotopic Stage (MIS) 5e [78]—to the present (Figure 9). Therefore, our response analysis will be restricted to this time interval, which also coincides with the Late Pleistocene. If future studies identify unequivocal paleobotanical evidence [20] of the oldest Caribbean mangroves, the analysis shall be extended backward accordingly.

During the last glacial cycle, Caribbean SSTs have followed trends similar to the global average, starting from interglacial (MIS 5e) temperatures higher than at present, followed by a further (MIS 5d) 4 °C decline to glacial temperatures. Full glacial conditions were recorded from about 110 to 20 kyr BP (Weichselian Glaciation), when minimum MIS 2/LGM (Last Glacial Maximum) temperatures were attained [79]. The record of land temperatures is limited to the last 45 kyr [80] and shows a higher amplitude of variation (>5 °C) between the LGM minimum and the present.

Moisture proxies displayed a high-frequency variability consisting of millennial-scale cycles correlated with the Dansgaard-Oeschger (D-O) events, which have been linked to periodic ocean circulation patterns [81,82,83] (Figure 9). In the Cariaco record, D-O cycles have been interpreted in terms of recurrent latitudinal migrations of the ITCZ leading to periodic shifts in rainfall from wetter (northward migration) to drier (southward migration) conditions [81]. In the Cuba record, rainfall variability seems to have been more linked to the Atlantic Meridional Overturning Circulation (AMOC), with wetter climates during phases of strong AMOC and drier periods under weak AMOC conditions [83]. Cold reversals such as Heinrich (H) events and the Younger Dryas (YD) have also been related, in the Caribbean region, with the southern ITCZ and weak AMOC, and hence, with drier climates. It is noteworthy that both moisture records show an abrupt shift toward drier climates during MIS 4 (D-O event 18); however, whereas Cariaco returned to wetter conditions shortly after, Cuba remained at low moisture values until the LGM.

Caribbean sea-level reconstructions for the last glacial cycle have largely concentrated on MIS-5 coral terraces, except for a continuous curve from the Gulf of Cariaco based on seismic stratigraphy [84,85,86,87,88,89] (Figure 9). These reconstructions show good agreement in documenting sea levels 3–20 m higher than at present during the Eemian interglacial (MIS 5e). However, inferences from coral terraces are consistently higher than those derived from seismic stratigraphy, as well as the estimated global averages for MIS 5a and MIS 5c. The most evident departure of seismic estimates with respect to the global curve occurs during the LGM, when the first are >40 m higher than the second. The Late Glacial and Holocene rising trends will be analyzed in more detail in the next section.

### 3.2. Late Glacial-Holocene Environmental Variability

**Figure 10:**
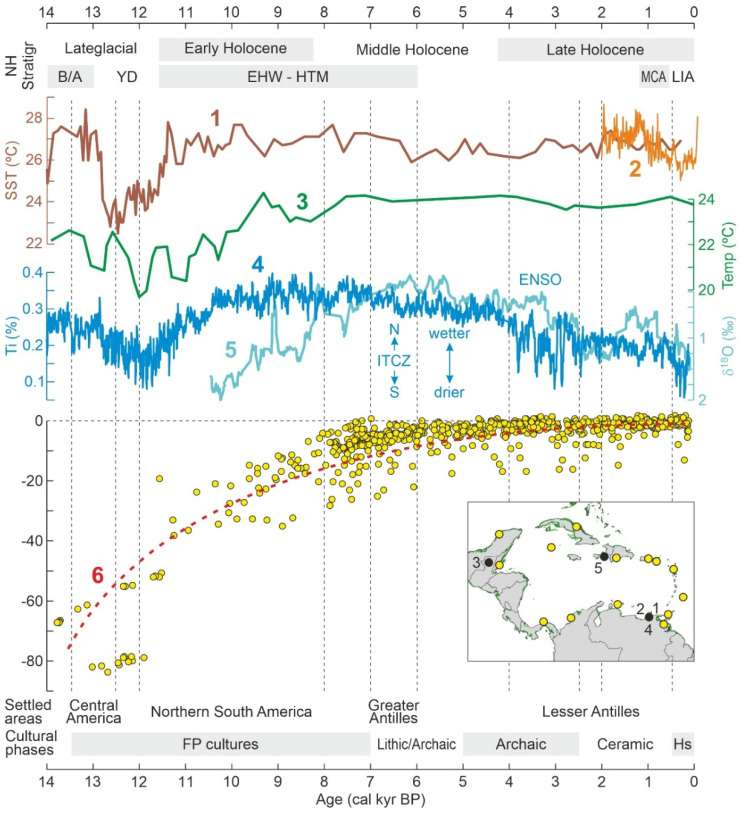
Late Glacial and Holocene temperature, moisture, sea-level changes and human settlement in the Caribbean region. (1) Sea Surface Temperature (SST) reconstruction from core PL07-39PC (Cariaco Basin, Venezuela) [95]; (2) High-resolution record of the last two millennia from core PL07-72GGC (Cariaco Basin, Venezuela) [96], vertical axis as in (1); (3) Land temperature reconstruction from Lake Petén Itzá (Guatemala) [80]; (4) Titanium (Ti) concentration as a proxy for continental moisture (river discharge) in core ODP-1002 (Cariaco Basin, Venezuela) [97]; (5) Ostracod oxygen isotope ratio from Lake Miragoane (Haiti) as a moisture proxy (the same ITCZ dynamics apply) [98]; (6) Sea-level trends, as deduced from almost 500 sites widespread across the Caribbean region (yellow dots in the reference map) [99]. B/A, Bølling/Allerød; Cr, Ceramic culture; EHW, Early Holocene Warming; Eu, Europeans; FP cultures, Fishtailed-Point cultures; G Antilles, Greater Antilles; Hs, Historical; HTM, Holocene Thermal Maximum; ITCZ, Intertropical Convergence Zone; LIA, Little Ice Age; MCA, Medieval Climate Anomaly; YD, Younger Dryas.

After the LGM, the classical Northern Hemisphere interstadial-stadial sequence Bølling/Allerød (B/A)-Younger Dryas (YD) was also recorded in both marine and terrestrial Caribbean paleo temperature reconstructions (Figure 10). In this case, the YD cooling was more intense and abrupt in the marine Cariaco record [80,95]. The Holocene began with the Early Holocene Warming (EHW), followed by the Holocene Thermal Maximum (HTM), which is also manifest in both records. A high-resolution record of the last two millennia [96] revealed the occurrence of climatic reversals consistent with the Medieval Climate Anomaly (MCA; 900–1280 CE) and the Little Ice Age (LIA; 1300–1890 CE).

Moisture shifts were recorded using the Titanium (Ti) concentration, a proxy for river discharge, and hence, continental rainfall. The Cariaco record suggested the dominance of wet climates for the B/A and the EHW-HTM and arid climates for the YD (Figure 10). As in the case of full glacial conditions, millennial-scale rainfall shifts would have been controlled by the latitudinal ITCZ migration. The onset of the Late Holocene (4000–3000 calibrated years before present, or cal yr BP) was characterized by century-scale variations in precipitation that have been linked with enhanced ENSO activity, specifically increases in the frequency of El Niño conditions. This phase is called here the Cariaco Holocene Instability (CHI) event and was punctuated by several extreme dry events similar in intensity to the YD but of much shorter (decadal- to multidecadal-scale) duration. A distinct drought event was recorded during the LIA, possibly related to the ITCZ migration [97]. Again, oxygen isotope records from Cuban speleothems encompassing the last millennia suggest that Atlantic circulation, in this case the Atlantic Multidecadal Oscillation (AMO), would be more influential than ITCZ migration in the northern Caribbean sector [100,101]. Other records provide support for the already mentioned climatic shifts, suggesting the occurrence of regional environmental trends [102]. A more heterogeneous picture seems to emerge for the Late Holocene, with higher hydroclimatic spatial variability for the last three millennia in Central American (Yucatán), Antillean (Cuba) and northern South American (Cariaco) sites [97,100,103]. However, the occurrence of last-millennium multiyear droughts in the Cariaco record, likely caused by the long-term ITCZ dynamics, have been considered of regional significance and intense enough to foster the cultural collapse of Central American pre-Columbian societies [104].

During the last 14,000 years, Caribbean sea levels experienced a maintained logarithmic-like increase with maximum rates of 7–11 m/1000 yr during the Late Glacial and the Early Holocene (up to 8200 cal yr BP), then declining to <2.5 m/1000 yr in the Middle Holocene (8200–4200 cal yr BP) and <0.5 m/1000 yr during the last 4200 yr. The relative sea level (RSL) did not exceed the present height during the Holocene, except in some northern South American localities in Guyana and Surinam, where it was around 1 m above the present by 7000 cal yr BP [99].

### 3.3. Human Settlement

#### 3.3.1. Late Pleistocene

**Figure 11:**
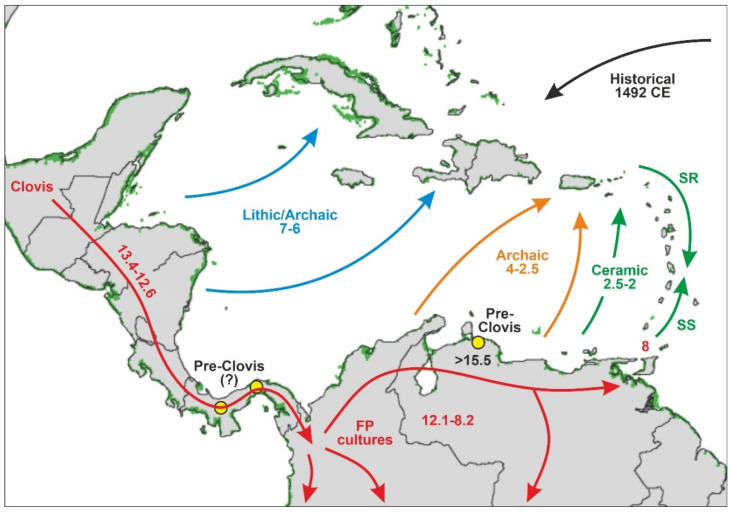
Human migration and settlement patterns across the Caribbean region considered in this study, composed from Refs. [105,109,110,111,114,115,116,117,118,119]. Numbers are ages in cal kyr BP. FP cultures, Fishtailed-Point cultures; SR, southern route hypothesis; SS, stepping-stone hypothesis.

One of the earliest records of human presence in South America was found on the southern Caribbean coasts, in the Venezuelan site of Taima-Taima, where Paleoindian artifacts dated at 17,300–15,500 cal yr BP were found associated with mastodon remains, suggesting megafaunal hunting [105] (Figure 11). This finding predated the YD cooling (Figure 9 and Figure 10) and the Clovis culture, which was the North American Paleoindian culture that crossed Central America, and rapidly expanded across South America between 13,000 and 12,500 cal yr BP [106,107]. It remains unclear, however, where pre-Clovis cultures originated and whether they used the Isthmian land bridge to reach South America [108]. Some evidence pointing toward this possibility was found in Panama, after the finding of some artifact fragments that were undated but stylistically similar to those of Taima-Taima [109,110,111]. However, most evidence from Central America corresponds to the Clovis southward expansion, dated at 13,400–12,600 cal yr BP [108]. Therefore, according to the available evidence, the first Caribbean places to be settled by humans were the South American and Central American coasts. After this first expansion wave, there is evidence of further occupations in Central and northern South America between the terminal Pleistocene and the Early Holocene, 12,100 to 8200 cal yr BP [108,112]. These Clovis-derived Paleoindian cultures were characterized by the so-called Fishtail Points (FPs) and are often referred to as FP users or FP cultures [107]. Noteworthy, SE Caribbean coasts were settled during the Early-Middle Holocene (up to 8000 cal yr BP), when the present island of Trinidad was still accessible by land due to lower sea levels [113].

Little is known about the natural resources used by Late-Pleistocene Clovis and earlier cultures, other than that they were foragers and active hunters and the now extinct megafaunal species that occupied the region by the time (mastodon, toxodon, glyptodon, ground sloth, giant deer, horse) were among their usual preys [108]. It has also been proposed that these cultures would have largely been responsible for continental-wide megafaunal extinctions [120]. From a paleoenvironmental perspective, human expansion into South America across the isthmian bridge coincided with the first half of the YD reversal, when climates were significantly colder and drier and the sea level was between 60 and 80 m below its present position (Figure 10), thus facilitating coastal migration through the exposed continental shelf. The FP consolidation across central and northern South America coincided with the EHW and the first half of the HTM, under climates that were increasingly warmer and wet, which would have favored the growth of human populations. However, no information is available in this regard.

#### 3.3.2. Holocene

The northern and eastern Caribbean coasts, corresponding to the Greater and Lesser Antilles, respectively, were settled later, in the Middle and Late Holocene, a time interval that has traditionally been subdivided into four main ages [121]: Lithic (7000–5000 cal yr BP), Archaic (5000–2500 cal yr BP), Ceramic (2500–500 cal yr BP), and Historical (1492 CE onward) (Figure 10). Others consider that archaeological evidence does not support the distinction between the Lithic and Archaic ages and merges them into a single age called Archaic or Preceramic [122,123]. In Mesoamerica—southern Mexico and northern Central America, where the Maya civilization developed and attained preeminence—the period between about 4000 and 1700 cal BP is known as the Preclassic or Formative period, whereas the Classic and Postclassic periods have been defined for the intervals 1700–1050 cal yr BP and 1050–430 cal yr BP, respectively. Interestingly, the ENSO-driven CHI climatic phase, as documented in the Cariaco record (Figure 10), coincided with the transition from the Archaic to the Mesoamerican Maya Preclassic/Formative period [124].

According to the prevailing view, the earliest colonization of the Greater Antilles dates back to ~7000–6000 cal yr BP, when Lithic/Archaic peoples arrived in Cuba and Hispaniola, probably from Central America [117] (Figure 11). Archaic peoples from South America occupied Puerto Rico and the northern Lesser Antilles by 4000–2500 cal yr BP. These islands were newly settled by Ceramic cultures, also of South American origin, between 2500 and 2000 cal yr BP [119]. In this case, however, two alternative hypotheses have been suggested. The first, called the stepping-stone (SS) hypothesis, proposes that the Lesser Antilles were colonized from South America following a northward pathway, whereas the second, the southward route (SR) hypothesis, contends that the Lesser Antilles were settled from the previously colonized northern Antilles [119].

Information on subsistence strategies is also scarce for the Lithic and Archaic phases. Contrary to earlier views, there is mounting evidence that Archaic Caribbean peoples were not simply nomadic foragers using coastal resources but managed and possibly cultivated a number of plants, notably maize in Mesoamerican cultures, lived in permanent villages, and regularly made and used pottery. Anthropogenic fires seem to have been the main environmental modifier during Archaic times [117]. Environmentally, Preceramic cultures developed under Mid-Late Holocene warm climates, with a long-term trend toward drier conditions and sea levels slightly below present ones (Figure 10).

More information is available for Ceramic cultures (2500–1400 cal yr BP), which inhabited primarily, but not exclusively, coastal environments. Subsistence strategies involved hunting of land and sea animals, fishing, collecting shellfish and cultivation of root crops, notably manioc (*Manihot esculenta*), which was introduced to the Caribbean islands by these cultures, and a number of different fruits, tubers and seeds [118]. The introduction of ceramic technology linked to manioc cooking and processing was also a relevant cultural contribution. Marine foods constituted the most important part of the diet, as the terrestrial fauna was rather impoverished. Fishing technologies included hook and line, spears, traps, poison and nets [117].

A major social and environmental transition took place since 1000 cal yr BP onward, probably linked to a shift from drier to wetter climates and higher sea levels, which would have enhanced agricultural potential and the distribution of near-shore environments. A deep change in pottery styles has been documented for this phase, although subsistence practices seem to have remained more or less constant [125,126]. However, some additions to the diet, such as a wild variety of sweet potato (*Ipomoea* sp.) were significant [127]. In general, the trend during the Ceramic Age was the expansion of Amerindian groups from South America into virtually every island group in the Caribbean (with the exception of the Cayman Islands), the introduction of several new species of plants and animals, a general decrease or extermination of several indigenous species of fish, crabs, rats and birds on many islands, and an increased reliance on terrestrial and/or horticulturally important foods that required the clearance of forests [117].

## 4. Mangrove Paleoecology

### 4.1. Modern Pollen Sedimentation

**Figure 12:**
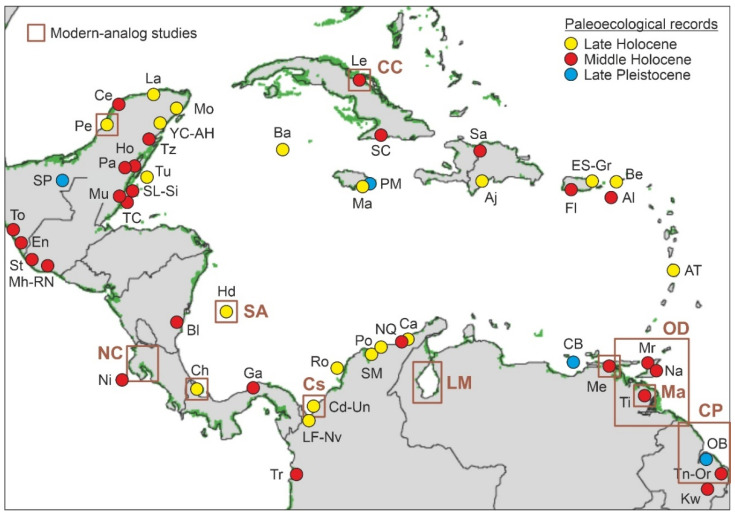
Circum-Caribbean sites and areas with Pleistocene–Holocene mangrove records (dots) and modern-analog pollen studies (boxes). Dot colors indicate the oldest age of each record. Abbreviations of paleoecological sites according to Table 3. Modern-analog study sites: CC, Cayo Coco (Cuba) [132]; Ch, Changuinola (Panama) [133]; CP, coastal plain (Guyana) [131]; Cs, Cispatá (Colombia) [134]; LM; Lake Maracaibo (Venezuela) [135]; Ma, Caño Macareo (Venezuela) [136]; Me, Playa Medina (Venezuela) [137,138]; NC, northwest Costa Rica [139,140]; OD, Orinoco delta (Venezuela) [130]; Pe, Petenes (Mexico) [141]; SA, San Andrés (Colombia) [142]; Ti, Caño Tigre (Venezuela) [143].

Accurate interpretations of past palynological evidence in terms of diversity, community composition, and spatial patterns largely rely on taphonomic knowledge, which is commonly addressed through modern analog studies using the principle of uniformitarianism [128]. Modern analog studies commonly compare current patterns of pollen sedimentation with the actual patterns and composition of the source plant communities to derive qualitative and/or quantitative relationships useful to infer past community features from sedimentary pollen records [129]. The circum-Caribbean region is in the roots of modern analog studies, as pioneering palynological surveys of this nature were developed in the Orinoco delta (Venezuela) and the Guyanan coasts about six decades ago [130,131]. The very first of these studies is still the most extensive (a hundred sites across >100,000 km^2^; 1 site every 1000 km^2^) developed in the Caribbean region and provided a thorough picture of pollen sedimentation patterns across the whole Orinoco delta and the adjacent shallow-marine environments [130]. Since then, a dozen Caribbean sites/areas have been studied for modern pollen analogs with paleoecological purposes (Figure 12).

An early review of modern mangrove pollen sedimentation along the Caribbean and Atlantic coasts of northern South America emphasized the following general points [19]:

- The better-represented mangrove trees in modern pollen assemblages are *Rhizophora* and *Avicennia*, whereas *Languncularia* and *Conocarpus* are usually scarce or absent.
- The pollen of *Rhizophora* is the most abundant by far, reaching values of 80% in the source area, and is commonly overrepresented in relation to the abundance of the parent species. This pollen declines landward but is still present in low percentages beyond the inland boundary of the mangrove fringe. In shallow marine environments, *Rhizophora* pollen may be found >80 km offshore in high percentages (50%) [130].
- The pollen of *Avicennia* is usually underrepresented with respect to its parent species, and its abundance rarely exceeds 40% of the pollen assemblage, even in pure stands of these three, remaining below 25% in mixed *Rhizophora-Avicennia* stands [131]. This pollen settles comparatively sooner, and its abundance rapidly declines in both inland and offshore directions [130].
- The pollen of *Conocarpus* is particularly difficult to find and may be absent even in the sediments of mangrove stands dominated by this tree [130,137]. The pollen of *Laguncularia* is rare.
- The spores of the mangrove fern *Acrostichum* are more abundant behind the mangrove fringe, close to the habitat of the species, and are present in low abundances in other mangrove soils. It may also be found in moderate abundance on the sediments of river mouths [135]. It has been suggested that *Acrostichum* could be indicative of human disturbance [137].
- These patterns are linked to interspecific differences in pollen production, transport, sedimentation and reworking processes that are discussed in the original references. Noteworthy, *Rhizophora* is wind-pollinated and produces large amounts of pollen [51], whereas *Avicennia*, *Laguncularia,* and *Conocarpus* are pollinated by insects and produce far less pollen [58,59,63].

The implications of these sedimentary patterns for the interpretation of past pollen records seem straightforward. First, *Rhizophora* pollen abundances tend to exaggerate the importance of its parent species, whereas *Avicennia* pollen amounts produce the opposite effect. Second, the absence/scarcity of *Conocarpus* and *Laguncularia* pollen is not indicative of the absence/scarcity of the parent species. It should be stressed, however, that these general points are useful to provide an overall regional-wide perspective on modern sedimentation patterns, but site-specific paleoecological interpretations should be addressed using the particular vegetation and depositional patterns of each locality or sedimentary domain.

Further works provided more information in this respect. Some studies along the SW Caribbean coasts typically involving depositional complexes around small bays and/or lagoons, have provided more detail on sedimentary subenvironments that are useful for paleoecological interpretation of local mangrove records. These surveys corroborated the sedimentary patterns of *Rhizophora* and *Avicennia* mentioned above and were able to locate the sedimentary signal of scarcer pollen types, such as *Laguncularia* and *Conocarpus*. For example, in some Panamanian and Colombian sites, *L*. *racemosa* is an important mangrove component and its pollen is common and abundant (up to 30%), especially in low-salinity environments, where this species establishes as a secondary colonizer of terrains submitted to anthropogenic exploitation of *Rhizophora*. Therefore, the finding of significant amounts of *Laguncularia* pollen in sediments may be considered and indicator of mangrove disturbance [134]. The same could be true for *Acrostichum*, which reinforces the observations of previous studies [137]. The scarcity of *Avicennia* and *Conocarpus* pollen (typically below 2%) in the same studies are explained in terms of low pollen production, but their presence could be indicative of saline back swamp (*Avicennia*) and supratidal (*Conocarpus*) environments. The indicator character of *Conocarpus* pollen is reinforced by its high abundance (up to 45%) in some supratidal environments submitted to low flooding intensity/frequency [134,142].

In the NW Caribbean sector, represented by the Mexican Yucatan Peninsula, paleoecological emphasis has traditionally been placed on inland forest records, and although modern-analog pollen studies are common [144,145,146,147], few of them include mangrove environments. A single study was found with a representative number of mangrove samples, which was aimed at differentiating modern pollen assemblages from several coastal and inland vegetation types [141]. This study concluded that mangrove vegetation was characterized by sedimentary pollen from the usual mangrove-tree species, but no further intra-community differentiation was attempted. The same is true for other Central American sites in Costa Rica [139,140]. Other Yucatan sites with modern analog studies have been used as statistical training sets for obtaining transfer functions to estimate paleoprecipitation patterns from pollen data, but no pollen-vegetation relationships have been established [146]. For obvious reasons, these paleoprecipitation reconstructions cannot be used to analyze the responses of the vegetation types used for calibration to moisture shifts.

In the northernmost circum-Caribbean area, a single study on modern pollen sedimentation was found in northern Cuba. There, *Avicennia* pollen is better represented in modern samples than in any other Caribbean sector, attaining values of 25% in stands dominated by this tree and 65% in hypersaline environments, where *A*. *germinans* occurs in association with the halophytic saltwort *Batis maritima* while *Rhizophora* is scarce or absent [132]. The pollen of *Laguncularia* and *Conocarpus* is scarce or absent across this Cuban study area, as it occurs on many northern South American coasts [19].

### 4.2. Paleoecological Sites

As mentioned before, quantitative palynological reconstructions constitute the main bulk of this review, although other types of evidence for the presence of mangroves have also been considered. Overall, the literature review carried out has identified a total of 59 sites with information useful to reconstruct Late Pleistocene and Holocene mangrove development across the Caribbean region (Table 3, Figure 12). Only four of these localities correspond to the Late Pleistocene (>11,700 cal yr BP), and only one, Ogle Bridge (Guyana), has an Early Holocene age (9700 cal yr BP). Most of the remaining 55 localities (50) contain Late Holocene (4200–0 cal yr BP) records, and 32 also include Middle Holocene (8200–4200 cal yr BP) sequences. There is no apparent geographical pattern in these chronologies.

**Table 3:** Caribbean mangrove records considered in this study. All records were dated by ^14^C, except for the youngest sections (dated by ^210^Pb) and the Pleistocene sections, which were dated by isotopic correlation (MD03-2622) and electron spin resonance (ESR) (P. Morant). Multiproxy studies always contain pollen records. Oldest ages (OA) of records, the first records of cultivated plants (CP), and the onset of significant/continuous charcoal records (Ch) are in cal yr BP (* yr BP beyond the ^14^C range), unless specifically stated. Dates have been taken from the original references and rounded to the nearest tenth. Coasts: CA, Central America; GA, Greater Antilles; LA, Lesser Antilles; NSA, northern South America. Country/island (C/I) codes are as in Figure 5 (SC, St. Croix). Proxies: A, archaeology (lithic/ceramic/shell/coral artifacts); Am, animal macroremains; Ch, charcoal; D, dinoflagellates; Dt, diatoms; F, foraminifers; G, geochemistry (organic matter, elemental analysis, stable isotopes); Mp, molecular phylogenetics and phylogeography; O, ostracods; P, pollen; Ph, phytoliths; Pm, plant macroremains; Py, physical properties (magnetic susceptibility, paleomagnetism, petrography/mineralogy); S, sedimentology/stratigraphy; St, starch; T, thecamoebians.

Site (core)	Map	C/I	Coast	OA	Proxies	CP	Ch	References
Urabá (Candelaria)	Cd	Co	NSA	100	P	-	-	[148]
Urabá (Uno)	Un	Co	NSA	100	P	-	-	[148]
Cispatá (Navío)	Nv	Co	NSA	200	P/G	-	-	[149]
Espíritu Santo (BC7)	ES	PR	GA	270	G/F/T	-	-	[150]
ERIA (Rosario)	Ro	Co	NSA	340	P	-	-	[151]
Cispatá (Flotante)	LF	Co	NSA	890	P/G	-	-	[149]
Alejandro (ALEJ08)	Aj	DR	GA	1000	P/Ch/G/Py	-	1000	[152]
S. Andrés (Honda)	Hd	Co	NSA	1100	P	1100	-	[153]
Manatee (MB-7)	Ma	Ja	GA	1200	O/G	-	-	[154]
Guajira (Calancala)	Ca	Co	NSA	1200	P	-	-	[155]
Anse Trabaud	AT	Mr	LA	1290	A/St/Pt/Am	1290	-	[156]
Pozo (Neguanje)	Po	Co	NSA	1800	P/G/Pm	-	-	[157]
Barkers (BA 1-9)	Ba	Cy	GA	2100	S	-	-	[158]
Santa Marta	SM	Co	NSA	2400	P/Py	-	-	[159]
Morelos (MPM-1)	Mo	Mx	CA	2520	P	-	-	[160]
R. Naranjo (OC001)	RN	Gu	CA	3050	P/Ch/Ph	3050	3050	[124]
Yax Chen	YC	Mx	CA	3500	S	-	-	[161]
L. Grande	Gr	PR	GA	3600	P/Ch/S/D/F/G	-	1550	[162]
R. Lagartos	La	Mx	CA	3870	P	3860	-	[146]
Petenes (Xpuk chico)	Pe	Mx	CA	4000	P/G/Py/S	2150	-	[163,164]
Unnamed (SC01)	SC	Cu	GA	4000	P/Ch/F/S	-	4000	[165]
Changuinola	Ch	Pa	CA	4000	P	-	-	[133]
Sipacate (SIP99E)	St	Gu	CA	4020	P/Ch/Ph	4000	3200	[124]
Altona (070321)	Al	SC	LA	4700	P/S/G	-	-	[166]
Tribugá	Tr	Co	NSA	5000	P/S	-	-	[167]
Palmar	Pa	Mx	CA	5000	P	-	-	[168]
Turneffe (TNF-1)	Tu	Bz	CA	5000	P/G	-	-	[169]
P. Galeta (GAL18)	Ga	Pa	CA	5200	P/G	-	-	[170]
Celestum (4A-4B)	Ce	Mx	CA	5300	F/G	-	-	[171]
R. Naranjo (TIL016)	RN	Gu	CA	5390	P/Ch/Ph	4620	4620	[124]
Bluefields (FBM1-5)	Bl	Ni	CA	5400	P	-	-	[172]
R. Hondo	Ho	Mx	CA	5600	P/G	5600	-	[173]
Sipacate (SIP014)	St	Gu	CA	5600	P/Ch/Ph	5520	5520	[124]
L. Flamenco (P01)	Fl	PR	GA	5660	G	-	-	[174,175]
Tonalá (La Joya)	To	Mx	CA	5600	P/Dt/G/Py	5600	-	[176]
Sipacate (SIP001)	St	Gu	CA	5820	P/Ch/Ph	5520	5520	[124]
Nariva	Na	TT	LA	5990	P	-	-	[177]
C. Tigre (A12)	Ti	Ve	NSA	6200	P/Ch/Py/G	-	6000	[143]
Encrucijada (C02)	En	Mx	CA	6200	P/Ch/F	4900	5300	[178]
Nicoya (DSDP-565)	Ni	CR	CA	6255	P	-	-	[139]
Guajira (N. Quebrado)	NQ	Co	NSA	6280	P	-	-	[155]
Sibun (SR-63)	Si	Bz	CA	6780	P/G	-	-	[179]
Manchón (MAN015)	Mh	Gu	CA	6780	P/Ch/Ph	-	5520	[124]
L. Leche (LL1)	Le	Cu	GA	6800	P/Pm/F/O/G	-	-	[180]
Aktun Ha	AH	Mx	CA	6830	P/G	-	-	[181]
Maracas	Mr	TT	LA	7000	P/G	-	-	[177,182]
Torani	Tn	Gy	NSA	7030	P/S	-	-	[131]
Oreala	Or	Gy	NSA	7370	P/S	-	-	[131]
Kwakwani	Kw	Gy	NSA	7390	P/S	-	-	[131]
P. Medina (PM-1)	Me	Ve	NSA	7790	P	-	-	[138]
L. Tzib	Tz	Mx	CA	7900	P/G	3500	-	[146]
Mullins-Sapodilla	Mu	Bz	CA	7980	S/G	-	-	[183]
Saladilla	Sa	DR	GA	8030	P/Ch/Dt	-	3600	[184]
S. Lookout (BT-79)	SL	Bz	CA	8100	P/S/G	-	-	[185]
Twin Cays	TC	Bz	CA	8200	S	-	-	[186]
Cariaco (MD03-2622)	CB	Ve	NSA	68 k *	P	-	-	[187,188]
S. Pedro	SP	Mx	CA	98 k *	Mp	-	-	[189]
Ogle Bridge (39)	OB	Gy	GA	>45 k *	P/Ch//S	-	Gl?	[131]
P. Morant	PM	Ja	GA	132 k *	S/Am	-	-	[77]

The Early Holocene gap is noteworthy and coincides with the FP expansion across northern South America (Figure 10 and Figure 11). As sea levels were 20–80 m below present ones (Figure 10), sedimentary evidence for Early Holocene mangroves, if present, should be far from present-day coasts under water depths of this magnitude. Indeed, records from the Middle Holocene are scarce before ~7000 cal yr BP, when sea levels attained heights similar to present ones. The sea-level effect is also evident in the Late Pleistocene, as the three available terrestrial mangrove records correspond to MIS 5, when sea levels were similar or above those at present, whereas the marine Cariaco record, whose sedimentary pollen originated in the adjacent South American coasts, corresponds to MIS 4-3, when sea levels were >40 m below (Figure 9).

### 4.3. Pleistocene

#### 4.3.1. Last Interglacial

The oldest well-dated Pleistocene evidence of in situ mangroves found in this review corresponds to the clastic-carbonate Jamaican Port Morant formation at Pera Point (Table 3; Figure 12), deposited in a lagoonal environment during the Last Interglacial. Within this formation, a sedimentary facies called VII is a fossiliferous sandstone that contains oysters with attachment scars of mangrove roots indicative of deltaic mangrove environments of normal marine salinities. An ESR (electron spin resonance) date on coral material from this facies yielded an age of 132 ± 7 kyr BP, which corresponds to the earliest highstand of MIS 5e [77], when sea levels were some meters above the present and SSTs were ~4 °C warmer than today (Figure 9). The lack of more precise taxonomic information hinders the establishment of more specific environmental relationships, but it is clear that climates that were significantly warmer and sea levels that were higher than present ones were still suitable for the development of Caribbean mangrove ecosystems.

Another Late Pleistocene record of similar age and environmental conditions has been documented for an interior Mexican locality from the south of the Yucatan Peninsula called San Pedro (Table 3; Figure 12). There, an inland mangrove community dominated by *R*. *mangle* was found growing far from coastal settings and saline waters [189]. The well-known wide environmental tolerance of this species makes possible its occurrence in this type of freshwater environment on calcium-rich substrates [190,191]. Molecular phylogenetic studies (using a mean generation time of 100 years and a median mutation rate of 3.16 × 10^−9^ mutations per nucleotide per generation) gave an estimated age of 98 kyr BP (confidence interval 5–273 kyr BP) for the divergence of the San Pedro mangroves and their closest sister coastal lineage, situated ~200 km north in SW Yucatan [189]. From these data, it has been inferred that the San Pedro mangroves could be a relic of Last Interglacial (Eemian or MIS 5e) coastal ecosystems, when sea levels were around this site, which is now 10 m above current sea levels. This would be consistent with the above Jamaican record and the general situation for the Caribbean region, as summarized in Figure 9.

#### 4.3.2. Last Glaciation

**Figure 13:**
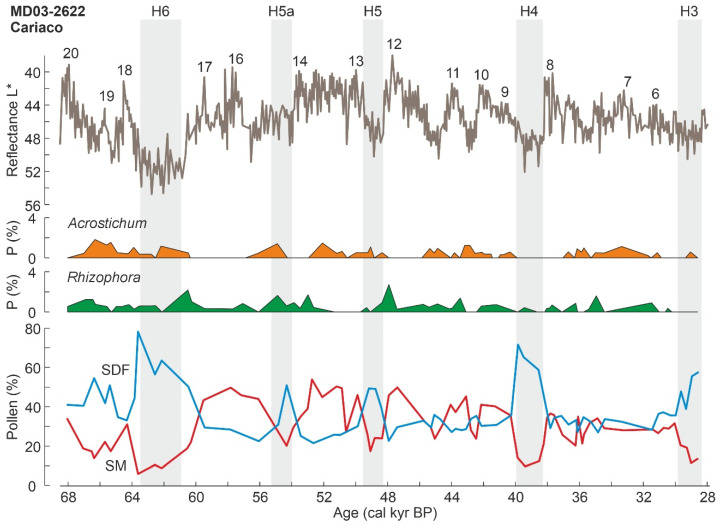
Cariaco pollen record from core MD03-2622 showing the changes in the abundance of mangrove elements (*Rhizophora* and *Acrostichum*), using the gray scale (reflectance) and the SDF (semi-deciduous forest) and the SM (salt marsh) percentage curves as a reference. H6 to H3 are Heinrich events, and numbers in the gray scale are D-O events. Composed from Refs. [187,188].

The third Late Pleistocene mangrove record reported here corresponds to glacial times, specifically the MIS 4/MIS 3 interval between approximately 70 and 30 kyr BP, which encompasses Heinrich events H6 to H3 and D-O events 20 to 6 (Figure 9). This record corresponds to the Cariaco Basin, one of the most emblematic sites for paleoclimatic reconstruction in the Caribbean region [74,75,81,97]. The palynological Cariaco record comes from a marine core (MD03-2622; Figure 9) located at a water depth of almost 900 m and has been used as a proxy for environmental and vegetation shifts occurring along the adjacent northern South American coasts, whose plant communities are the main pollen providers. An interesting finding was that salt marsh pollen underwent abrupt expansions during cold/dry Heinrich events (Figure 13), showing a typical and recurrent chenopod-grass-sedge succession [187]. This was called the Heinrich-event vegetation mode and was paralleled by characteristic reductions in forest assemblages and the total pollen input from the continent. An interstadial mode was also recorded during warm/wet D-O peaks, consisting of inverse pollen trends, notably the expansion of semi-deciduous and evergreen forests from the continental coastal range [188].

Among the mangrove elements discussed in this paper, *Rhizophora* and *Acrostichum* were always under 4% in the Cariaco record, whereas the others were absent or not reported. In the original reference [188], these mangrove components are included in the association called littoral vegetation, but here they are shown separately to analyze their trends in more detail. *Rhizophora* and *Acrostichum* consistently showed small values during H-events (Figure 13), suggesting that these abrupt cold/dry reversals accompanied by sea-level drops did not favor the development of mangroves in the littoral fringe. In addition, *Rhizophora* showed rapid, yet small, increases just after each H-event, which may be interpreted in terms of a rapid response to post-H warmings, followed by further declines. A positive association of mangrove elements with D-O warming is also noteworthy and would support this interpretation. Therefore, it may be inferred that mangrove communities underwent small expansions during warm/wet/highstand conditions and retractions under the opposite circumstances.

**Figure 14:**
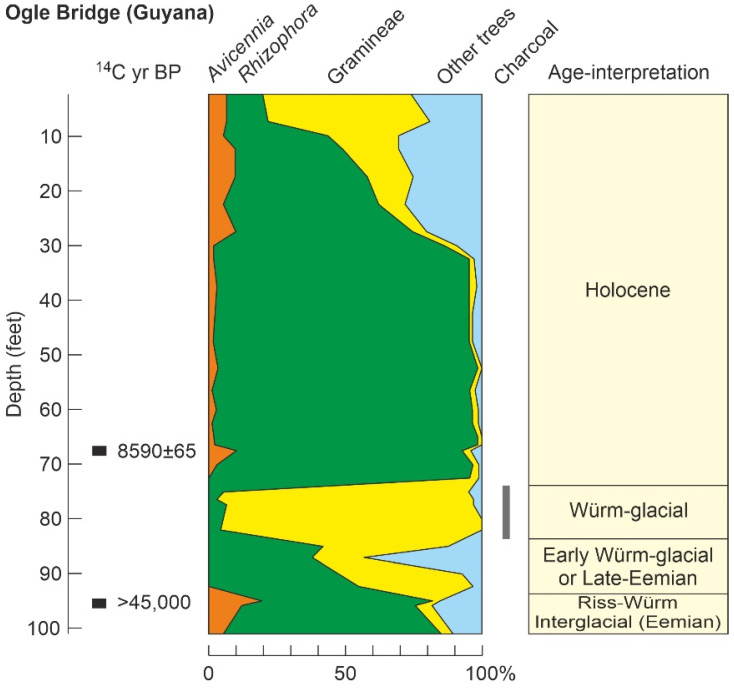
Summary pollen diagram from the Ogle Bridge section (Guyana) and chronological interpretation, according to the original Ref. [131]. Other trees include *Alnus*, *Ilex*, *Symphonia*, *Virola*, *Mauritia,* and other palms. Redrawn from Ref. [131].

The last Pleistocene record available, the Ogle Bridge record (Table 3; Figure 9), is from British Guiana (now Guyana) and consists of a sequence with poor chronological control that assumedly includes the Last Interglacial, the Last Glaciation, and the Holocene (Figure 14). This sequence has only two dates, one exceeding the ^14^C range and the other corresponding to the Early Holocene (8590 ± 65 ^14^C yr BP; ~9570 cal yr BP), which is the only date of this age found in this review. The chronological interpretation of the Ogle Bridge sequence largely relies on pollen stratigraphy [131]; therefore, attempts to analyze the responses of vegetation to climatic shifts would be circular. Noteworthy, the occurrence of the Last Glaciation is inferred from the dramatic decline of mangroves in favor of grass savannas, as deduced from pollen percentages in the interval between about 74′ and 84′. Therefore, arguing that mangroves severely contracted and savannas dramatically expanded due cold glacial conditions would be a logical fallacy. Independent support to this interpretation, however, may be found in the already discussed examples from Jamaica, Mexico and the Venezuelan Cariaco Basin, where warm/wet climates and high sea levels are commonly associated with mangrove expansions, whereas cold/dry and lowstand conditions lead to mangrove declines in favor of other coastal communities.

Another drawback of the Ogle Bridge section is that the few samples dated are not enough to detect eventual sedimentary gaps, which are common in coastal environments, especially under oscillating sea levels, as was the case for the last glacial cycle (Figure 9 and Figure 10). The occurrence of such gaps could help explain why the Holocene is about 70′ deep, whereas the Eemian interglacial and the Würm glaciation together, which lasted 10 times longer than the Holocene, are condensed into only 30′ (Figure 14). Noteworthy, a significant information gap of roughly 20,000 yr exists between the last well-dated Pleistocene (~28,000 yr BP) and the first Holocene (~8000 yr BP) Caribbean mangrove records (Table 3). This gap includes significant cool/dry events such as the LGM and the YD, whose paleoenvironmental features are reasonably well known (Figure 9), but the corresponding mangrove responses remain unknown. The Ogle Bridge sequence is the only one among those analyzed here that could contain evidence on LGM mangroves, but unfortunately, the discussed dating problems prevent more detailed conclusions. In the Cariaco Basin, some preliminary pollen data on the YD were released as a congress abstract [192], but no publication of the full record followed.

### 4.4. Holocene

**Figure 15:**
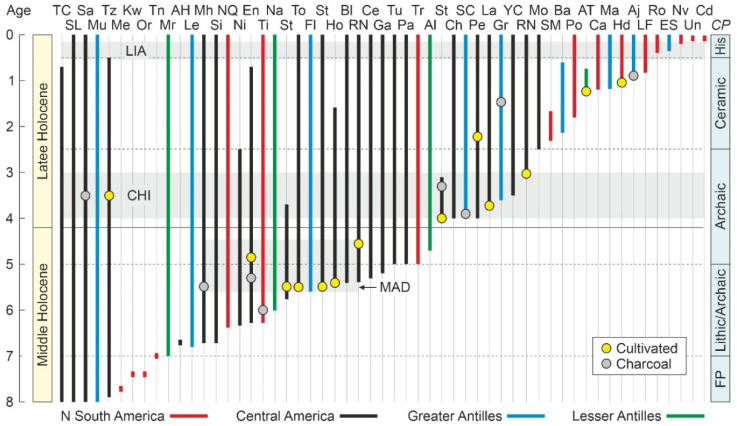
Holocene pollen records sorted by oldest date (time scale in cal yr BP). The different continental and insular Caribbean coasts are indicated by line colors. Yellow dots indicate the first appearances of pollen/phytoliths from cultivated plants, and gray dots mark the onset of significant/continuous charcoal records. Data and abbreviations from Table 3 (see Figure 12 for location). CHI, Cariaco Holocene Instability event; *CP*, Cultural Phases; His, Historical period; MAD, Mesoamerican Archaic Disturbance event.

**Figure 16:**
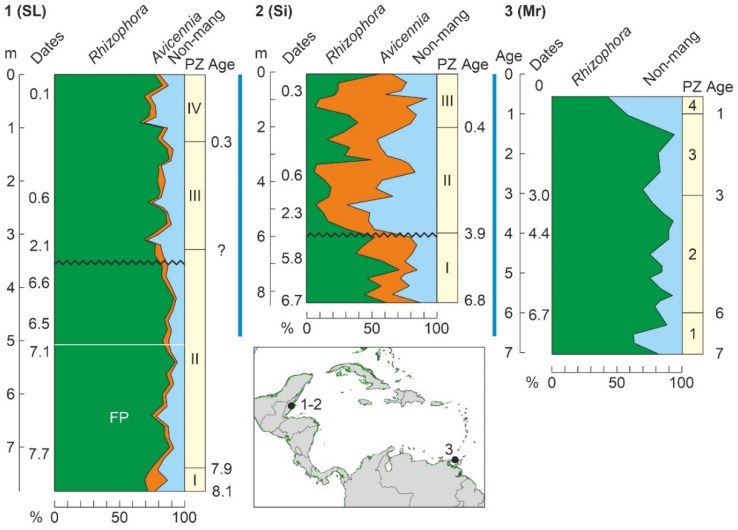
Summary pollen diagrams for the oldest and most complete mangrove records identified in this review. Diagrams SL and Si are plotted by depth (m), and Mr is plotted by time (cal kyr BP), as in the original references. All dates (calibrated from ^14^C dates) and ages (interpolated/extrapolated from age-depth models) are in cal kyr BP and have been taken from the original references. The broken line between six and two cal kyr BP in sites SL and Si indicates the approximate position of the millennial-scale sedimentary gap present in these sequences; the question mark in SL indicates the uncertainty of the II/III boundary due to the sedimentary gap. FP, Fishtailed Point cultures; PZ, pollen zones. Redrawn and composed from Refs. [179,182,185].

As discussed above, Holocene mangrove records begin at ~8000 cal yr BP (Middle Holocene), when temperatures were similar to today and sea levels were slightly below their present position (Figure 10). This also occurred when the continental Caribbean coasts were already occupied by FP cultures and shortly before the settlement of the Greater Antilles by Lithic/Archaic peoples (Figure 11). The oldest and most complete Holocene mangrove record corresponds to a Central American site (SL, Belize), which accounts for the last ~8000 yr [185]. Other fairly complete and well-dated records encompassing the last ~7000 yr are from sites Si [179], also in Belize, and Mr, in Trinidad [182] (Table 3, Figure 15). The main drawbacks of these sequences are the presence of a huge sedimentary gap between about 6000 and 1000 cal yr BP (SL and Si) and the low resolution (multicentennial) of the Mr pollen record (Figure 16).

A quick look at the diagrams in Figure 16 is enough to realize the high degree of spatial heterogeneity in Holocene mangrove composition, even in nearby sites such as SL and Si, which are only ~20 km apart. These differences in local mangrove composition are also evident today. Indeed, extant mangroves at SL are largely dominated by *R*. *mangle*, with *A*. *germinans* and *L*. *racemosa* present [185], whereas in Si mangrove forests, which are in a more inland position, *A*. *germinans* is more abundant than in the outer fringe [179], and both *A*. *germinans* and *L*. *racemosa* are absent in Mr mangrove communities [182]. This indicates that mangrove pollen records are highly sensitive to the zone of the sea-land transect where the coring site is located. Interestingly, these general compositional patterns have been more or less constant, with minor oscillations, during the Middle and Late Holocene sequences analyzed (Figure 16).

Other sequences (Mu, Sa, TC, and Tz) encompass similar time periods (Figure 15), but their chronological control is less robust or they are situated in more inland environments, and consequently, mangrove pollen is scarce and/or discontinuous throughout the sequences. Therefore, a complete and well-dated Mid-Late Holocene record of mangrove succession at centennial or lower resolution is unavailable, and the analysis of Holocene mangrove succession should be based on the composition of shorter records.

#### 4.4.1. Middle Holocene

The only well-dated record containing the FP period (>7000 cal yr BP) is SL (Figure 16). The interval of interest includes Pollen Zone I (PZ-I) and the lower half of PZ-II [185]. PZ-I (8100–7900 cal yr BP) represents the inception of mangroves when rising sea levels reached the site, suggesting that sea level was 8 m below the present by 8000 cal yr BP, which is consistent with regional reconstructions (Figure 10). Similar evidence for early colonization of Caribbean coasts by mangroves linked to sea-level rise is available for site TC, also from Belize [186], and some northern South American sites, such as Me, from Venezuela [138], and Kw, Or and Tn, from Guyana [131]. In the SL record, the first mangrove communities were dominated by *Rhizophora*, whereas *Avicennia* was present and *Laguncularia* absent. Mangroves fully established in the site during PZ-II (7900–4200 cal yr BP) and *Rhizophora* slightly expanded at the expense of *Avicennia*, which experienced a small but significant decline and never recovered their former PZ-I abundances. Although this is not mentioned in the original reference, this pattern may suggest an important contribution of *Avicennia* in the first colonization stages, which is supported by the well-known pioneering character of *A*. *germinans* in the upper intertidal fringe [58]. Regarding climate, the regional HTM precipitation maximum characteristic of the Caribbean region linked to the northern migration of the ITCZ (Figure 10) was also documented in the SL section using independent oxygen isotopic evidence [185]. Therefore, full mangrove development during PZ-II was favored by highstand conditions and wetter climates.

A well-dated record of mangrove response to sea-level rise during the last 7000 years is from the Orinoco Delta site Ti, which is currently ~20 km inland and does not support mangrove vegetation but palm-swamp freshwater stands dominated by *Mauritia* [143]. The situation was different before 5200 cal yr BP, when *Rhizophora* occurred at the site, which was closer to the intertidal zone, when sea levels were lower than today, as demonstrated by independent evidence from nearby sites [138]. This strongly contrasts with the situation in other Caribbean localities, where the occurrence of Holocene mangroves is commonly associated with increasing rather than decreasing sea levels. In these cases, it is considered that mangroves followed the eustatically-controlled altitudinal displacements of the intertidal zone, whereas in the Ti site, rising sea levels are more linked to lateral shifts that moved the delta-front coasts seaward, away from the coring locality. This is due to the fact that the Orinoco megadelta receives huge amounts of sediment from the mighty watershed of this river, which causes rapid coastal progradation and therefore lateral shifts of intertidal environments to be comparatively much faster than altitudinal sea-level displacements [193].

Until 6000–5500 cal yr BP, climate (notably moisture) and eustatic shifts were the main drivers of mangrove changes, but since those dates, a new element, humans, came into play. Human impact on Caribbean mangroves is commonly deduced from evidence independent of proxies used to record vegetation changes, as is the case with the occurrence of pollen or phytoliths from cultivated plants and/or significant increases in sedimentary charcoal [124]. In the Caribbean region, the most important Holocene cultivated plant was maize (*Zea mays*), which is known to have been used by Mesoamerican lowland peoples since before 5000 cal yr BP [194]. Another relevant cultigen was squash (*Cucurbita* spp.), which is usually inferred from the occurrence of Cucurbitaceae pollen [178]. In the Neotropics, the consistent occurrence of high charcoal amounts in Late Pleistocene and Holocene sediments has demonstrated to be a strong indicator of human presence [195,196]. In contrast to pollen from cultivated plants, which has been commonly identified since the very beginning of Holocene pollen analysis [197], the use of charcoal records as a fire proxy is more recent and less common. Therefore, the absence of charcoal in past records is not necessarily indicative of the lack of human activities in a given site.

In the localities considered here, the earliest records of pollen from cultivated plants date from 5500–5600 cal yr BP (sites Ho, To, and St, in Mesoamerica), which are among the oldest records for the region [124,173,176]. Regarding charcoal, the oldest records compatible with human presence are also of similar ages and come from the same region (sites St and Mh), with the exception of a locality (Ti) from the Orinoco Delta, which is the earliest (~6000 cal yr BP) of these records found to date [143]. The combination of the above evidence points to a first event of human disturbance on mangroves between 5600 and 4500 cal yr BP, in the transition between Lithic and Archaic ages (or around the middle of the Archaic Age, if the Lithic Age is not considered valid), which is called here the Mesoamerican Archaic Disturbance (MAD) event (Figure 15). Just before this event, between 6000 and 5400 cal yr BP, a phase of strong charcoal increase was recorded in the SE Caribbean region (Ti site, in the Orinoco Delta), which was attributed to the arrival of the first human colonizers [143] and would be the earliest evidence of human presence during the Holocene. The origin of these eventual human settlers is still unknown, but under the general scenario for human occupation of northern South America (Figure 11), it is possible that they were related to FP cultures.

**Figure 17:**
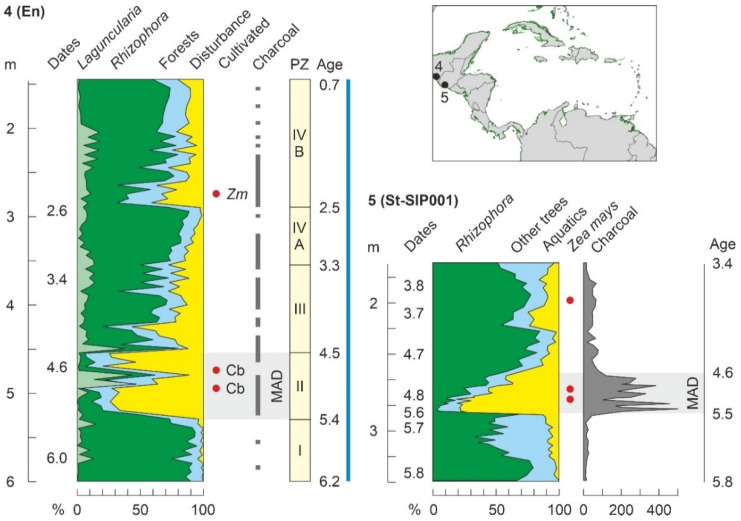
Summary subcentennial pollen records from Mesoamerica showing the conspicuous Mesoamerican Archaic Disturbance event (MAD) (gray band). The occurrence of pollen from cultivated plants is indicated by red dots (Cb, Cucurbitaceae; *Zm*, *Zea mays*). All dates (calibrated from ^14^C dates) and ages (interpolated/extrapolated from age-depth models) are in cal kyr BP and have been taken from the original references. Charcoal is represented in concentration units (particles/mL of sediment). PZ, pollen zones. Redrawn and composed from Refs. [124,178].

Well-dated subcentennial MAD records are available, for example, at sites En and St, from southern Mexico and Guatemala, respectively (Figure 17). In En, this event is well documented between about 5400 and 4500 cal yr BP, when a sudden increase of disturbance indicators, notably Chenopodiaceae/Amarantheceae, coincided with a dramatic reduction of *Rhizophora* in favor of *Laguncularia*, which is an active colonizer of disturbed *Rhizophora* mangroves [134]. This abrupt vegetation change was accompanied by the first appearances of Cucurbitaceae pollen and the onset of continuous charcoal records (unfortunately, only presence/absence was recorded in this case). The whole picture strongly suggests landscape anthropization and the initiation of agricultural practices [178].

**Figure 18:**
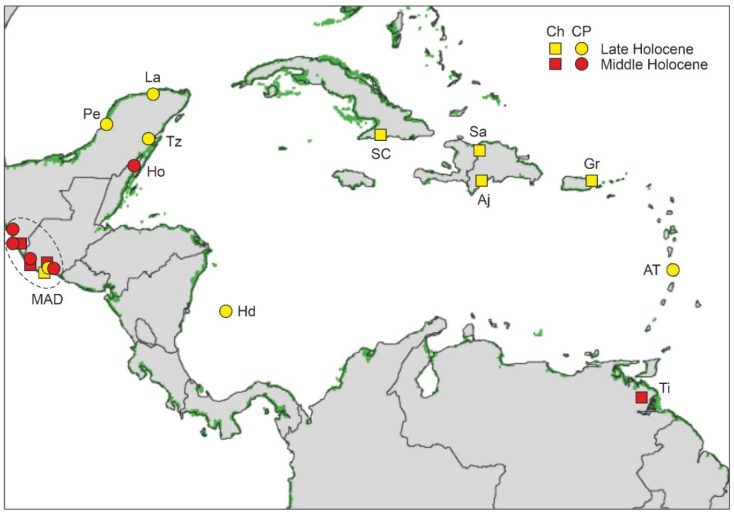
First appearances of pollen from cultivated plants (*Zea mays* and Cucurbitaceae) and charcoal amounts/patterns compatible with anthropogenic fires in Caribbean mangrove records. Raw data from Table 3 (see also Figure 15). Ch, charcoal; CP, cultivated plants; MAD, Mesoamerican Archaic Disturbance event.

The MAD event is also evident in the St record (core SIP001) between approximately 5500 and 4600 cal yr BP, this time with aquatic taxa (Cyperaceae, *Typha*) as secondary colonizers after mangrove clearing and maize as the first cultivated plant to be recorded (Figure 17). In this case, quantitative charcoal estimates (particles/mL of sediment) provided a better appraisal of the magnitude of local anthropogenic fires with respect to the background signal. Other nearby sites (RN, Mh, and other St cores; Table 3; Figure 12) show analogous patterns at similar time intervals [124], suggesting that the MAD event was more than a local event, although, according to the available evidence, it was restricted to the present Guatemala-Mexico border area (Figure 18). Indeed, in other records from nearby regions, for example the Yucatán site Ho, no similar mangrove disturbance has been documented, despite the continuous occurrence of significant amounts of maize pollen throughout the whole section (5600–1700 cal yr BP) [173]. People developing these practices were not merely foragers harvesting shellfish and other coastal resources but also slash and burn farmers [198]. These cultures were probably the origin of the people that first colonized the Greater Antilles [116] (Figure 11).

In other localities, human activities, although present, were less influential and mangrove dynamics were more controlled by climate, especially precipitation shifts. This is the case at the above-mentioned Yucatan Ho site, where independent geochemical paleoclimatic evidence suggests that, during the MAD phase, the ITCZ was displaced to the north, thus causing precipitation increases that favored the development of inland rainforests and freshwater wetlands, coinciding with a phase of minimum mangrove cover [178]. Other natural phenomena that affected mangroves were hurricanes and landfalls, which experienced a phase of quietness between 5400 and 4900 cal yr BP, coinciding with lower sea levels, the dominance of freshwater wetlands and reduced mangrove extent on the Nicaraguan coasts (site Bl), in the absence of evident human pressure [172]. In general, evidence for Mid-Holocene human disturbance is meager or nonexistent outside Mesoamerica, and natural environmental drivers, primarily eustatic shifts and moisture fluctuations seem to have been dominant across the Caribbean region.

#### 4.4.2. Late Holocene

As mentioned above, the Late Holocene began with a widespread and intense climatic reversal, the CHI, and a gradual increase of evidence for anthropogenic impact in both time and space (Figure 15). During the last 4200 cal yr BP, pollen from cultivated plants and charcoal increases compatible with human disturbance spread across the whole Caribbean region, including the Antilles (Figure 18), which had already been colonized by humans (Figure 11). Noteworthy, this type of evidence is consistently absent from southern and western Caribbean coasts, but whether this is due to the lack of human influence on mangroves or to the lack of charcoal studies—skipping maize and/or squash pollen in routine pollen analyses is unlikely—remains unknown. Therefore, during the Late Holocene, both natural and anthropogenic factors, along with their eventual feedbacks and synergies, should be considered as potential drivers of ecological change in mangroves across the whole Caribbean region. Again, spatial heterogeneity is evident in the effects of climatic, eustatic and anthropogenic factors. For example, during the CHI phase (4000–3000 cal yr BP), regional moisture shifts have been considered the main drivers of mangrove change in a Yucatan locality (Tu), whereas human disturbance has been suggested as the most likely cause for mangrove change in the Mesoamerican site St [124,169].

**Figure 19:**
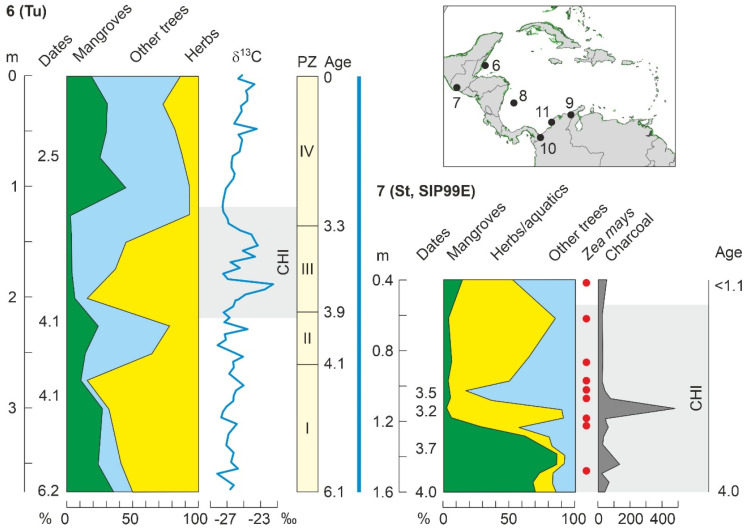
Summary pollen diagrams from Mesoamerican sites showing mangrove reductions around the CHI climatic event (gray band). The occurrence of pollen from cultivated plants is indicated by red dots (Cb, Cucurbitaceae; *Zm*, *Zea mays*). All dates (calibrated from ^14^C dates) and ages (interpolated/extrapolated from age-depth models) are in cal kyr BP and have been taken from the original references. Charcoal is represented in concentration units (particles/mL of sediment). PZ, pollen zones. Redrawn and composed from Refs. [124,169].

Pollen records from the first two localities (Tu and St) are of low resolution, but the interval of interest in this case is well constrained chronologically by radiocarbon dates (Figure 19). At the beginning of this climatic event, the diagrams of both sites show a drastic reduction of mangroves, which were replaced by terrestrial and aquatic herbs. However, despite their similarity, these vegetation shifts were driven by different factors in the two localities. In Tu, the phase of *Rhizophora* mangrove decline (Pollen Zone III, 3900–3300 cal yr BP) coincided with an interval of higher δ^13^C values, interpreted in terms of higher salinity resulting from increased evaporation rates under drier climates, which is consistent with local climate reconstructions [169] and with the Caribbean-wide CHI shift documented in the Cariaco Basin [97]. In support of this, no evidence of human disturbance, in the form of pollen from cultivated plants or charcoal increases, was found. Contrastingly, at the St site, the *Rhizophora* mangrove recession coincided with a conspicuous charcoal peak and the intensification of maize cultivation (Figure 19), and was considered to be the result of a second Mesoamerican anthropization phase at the beginning of the Preclassic Maya period [124]. However, the chronological coincidence between this anthropization event and the regional CHI drying trend is noteworthy and may suggest the occurrence of eventual climate-human feedbacks that could have amplified mangrove decline.

In other areas, mangroves remained relatively stable, despite the occurrence of regional-scale climatic and eustatic fluctuations. This is the case, for example, at the Panamnian site of Ga, where mangroves did not experience significant changes during the last 5000 years. These mangroves retained their extent and relatively constant abundances of their main components (*Rhizophora*, *Avicennia, Acrostichum*) throughout this Holocene interval, likely due to the interplay between climate, sea-level increase, and lateral coastal migration [170]. At the beginning of the Ga record (5200–5000 cal yr BP), mangroves were migrating landward due to the progressive sea-level rise, which was 4–5 m below its present position. A second stage (5000–3100 cal yr BP) was characterized by mangrove recession, which was interpreted as the result of intensified landward migration, probably due to drier climates, notably the CHI event, caused by the coupled action of ITCZ migration and ENSO intensification. This was supported by lower negative δ^13^C values and higher C/N ratios, which indicated the predominance of marine organic sedimentation. After a phase of maximum sea levels and minimum terrigenous sediment input (3000–1800 cal yr BP), mangrove pollen increased in the site between 1800 and 700 cal yr BP, most likely due to coastal progradation caused by increasing terrigenous input, favored by wetter climates. Proxies for anthropogenic disturbance were absent throughout this sequence.

This indicates that constancy in mangrove cover and community composition does not necessarily imply environmental stability and, once again, warns against the use of vegetation proxies for reconstructing environmental shifts aimed at studying the responses of plant communities to climatic and eustatic drivers. This case study and others from the Orinoco Delta and the NW Colombian coasts (sites Ti and NQ, respectively [143,155]) suggest that Holocene mangrove records are strongly influenced by the balance between terrigenous input—which is largely dependent on the magnitude of incoming fluvial systems and continental precipitation—and sea-level rise. During drier climatic phases, sea-level increase is dominant and mangrove communities migrate landward, whereas during rainy phases, sedimentation is able to compensate for or surpass eustatic rise and seaward coastal migration dominates. Once more, care should be taken in the type of evidence used to record mangrove responses to climatic and sea level trends, which should be inferred from pollen-independent proxies to avoid circularity. For example, it is not possible to infer sea-level trends from mangrove dynamics and then try to analyze the response of mangroves to the eustatic fluctuations deduced in this way.

During the last 3000 years, no significant regional environmental reversals have been documented in the Caribbean region beyond the already mentioned sustained drying trend (Figure 10). Anthropogenic disturbance intensified and spread across the Caribbean region to become a major environmental driver of mangrove dynamics (Figure 18). An example of human impact can be seen in the already discussed En site since 2500 cal yr BP, when *Zea mays* pollen reappeared in the diagram and charcoal records were continuous, coinciding with an abrupt mangrove decline and a significant increase in disturbance vegetation (Figure 17). Similar trends were observed in some St sections during the Preclassic Maya period [124].

Other records of mangrove reduction during the last three millennia have been interpreted in terms of climate change or climate-human interactions. For example, a significant *Rhizophora* decline occurred in site Gr (Puerto Rico) between 1550 and 575 cal yr BP was linked to a phase of greater tropical storm activity [162]. This mangrove recession also coincided with an outstanding charcoal peak that was considered to be indicative of human presence. Despite the lack of pollen from cultivated plants, this interpretation was supported by the finding of human-made artifacts dating back ~2000 cal yr BP (Ceramic age) around the coring site. A similar *Rhizophora* reduction accompanied by a significant increase in Amaranthaceae—which would have included halophytic species and others indicative of dry conditions and disturbance—was documented in Hispaniola since 2500 cal yr BP and was interpreted in terms of drier climatic conditions [184]. The occurrence, in the same record, of a charcoal peak by 1500 cal yr BP was attributed to increased fire activity due to dry climates, rather than human activities, due to the absence of indicators of human inhabitants around the region prior to European contact. In some records of similar ages from northern South America, mangrove declines were also associated with dry climates and further recoveries with wet conditions, as is the case at the Colombian site NQ [155].

#### 4.4.3. Last Millennium

Although part of the Late Holocene, the last millennium is discussed separately because of its peculiar features in both climatic and cultural terms. Climatically, the most relevant event was the LIA that, in the Caribbean region, was characterized by cool and dry climates [96,97] (Figure 10). Culturally, the main disruption was European contact and its subsequent consequences, which profoundly changed the patterns and intensity of land use and ecosystem disturbance.

**Figure 20:**
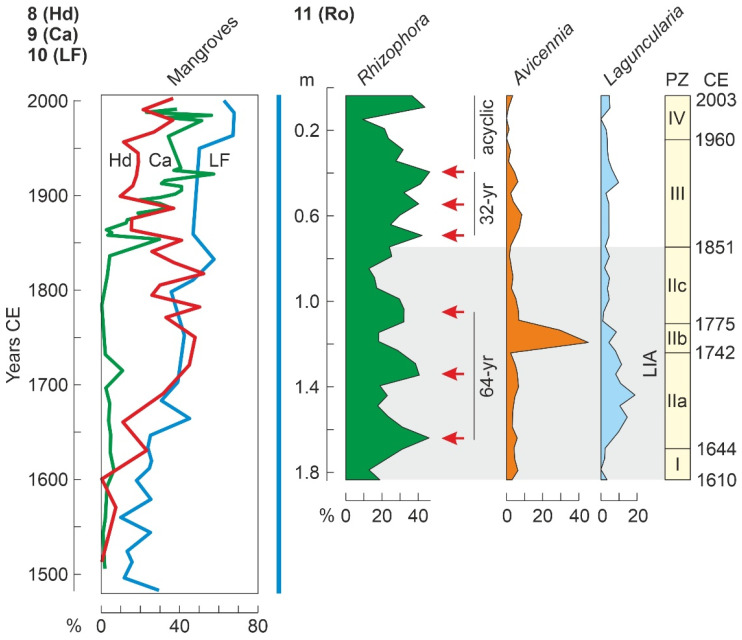
Mangrove dynamics on the Colombian Caribbean coasts during the last five centuries. See Figure 19 for the location of coring sites. PZ, pollen zones. Redrawn from Refs. [149,151,153,155].

Several high-resolution (bidecadal) records encompassing the last millennium are available for northern South American coasts and adjacent islands at the Colombian sites Ca, LF and Hd. In these records, mangroves were consistently scarce or absent at the beginning and increased or appeared only in the last few centuries. In LF, a gradual expansion took place between 450 and 100 cal yr BP, when mangroves attained their full development [149]. In Ca, modern mangrove communities were also established in the last 100 years, but they had been virtually absent until then [155]. A different pattern was observed in Hd, where mangrove communities gradually expanded between 400 and 200 cal yr BP, when they attained a maximum and initiated a sustained declining trend [153] (Figure 20). These patterns strongly contrast with another nearby site (NQ), where mangroves experienced a millennial-scale decline since 2500–2000 cal yr BP and almost disappeared during the last 500 years [155]. Such spatial heterogeneity in such a reduced area (Figure 12) has been explained by local differences—especially in geomorphology, precipitation/evaporation rates, sediment input from rivers, and human disturbance—which is considered to be a serious drawback for the definition of a mangrove-based regional eustatic curve [155].

Among climatic factors, extreme droughts during the LIA have been considered major drivers of mangrove reduction, probably due to hypersalinization and/or reduced sediment input and coastal progradation, which would have deteriorated the environment suitable for mangrove development [155]. Extreme meteorological events, notably hurricanes and destructive storms, could have also affected mangrove communities. For example, on site Hd, a phase of increased storm activity recorded in historical documents was considered to be responsible for a >2000-yr sedimentary gap before 1600 CE [153]. Regarding human activities, deforestation for a diversity of uses and exploitation practices seems to have been the most widespread activity. This is evident, for example, in the Hd pollen record, where a strong decrease of *Rhizophora* by 1850 CE coincided with the increase of *Cocos nucifera* (coconut), which is known to have been extensively cultivated at the site and the adjacent coasts since that date [151,153].

In addition, on the Colombian coasts, the Ro record provided more detail on the influence of climate on mangrove development during the last 400 years. A general conclusion was that climate influenced regional mangrove trends, whereas other factors were more local. In this record, *Rhizophora* pollen peaks exhibited periodic trends correlated with the Pacific Decadal Oscillation (PDO), the AMO, and the ENSO during the LIA (64-yr cycles), and between the mid-19th and mid-20th centuries (32-yr cycles) (Figure 20). Beyond 1954 CE, no significant cyclicity was found in the *Rhizophora* record, likely due to increasing human disturbance, mainly for wood extraction, fisheries, coconut plantations and rice crops. These mangroves were found to be resilient to climatic cycles, but showed low recovery rates to extreme/prolonged droughts and intense human pressure [151]. In general, extreme meteorological events (hurricanes, storms), coastal erosion/progradation, drought, salinization and human disturbance have been considered key factors for mangrove development during the last few centuries. These drivers have led to mangrove expansion (progradation) or reduction (erosion), as well as changes in their composition, notably the replacement of *Rhizophora* by *Avicennia* dominance under high-salinity conditions and increases in *Laguncularia*, *Acrostichum* and halophytic grasses under anthropogenic stresses [148].

In the Greater Antilles, hurricane activity profoundly affected mangrove communities during the last millennium, as recorded in site Aj from the Dominican Republic (Hispaniola). During the MCA, relatively moist climates would have facilitated mangrove recovery after hurricane disturbances, whereas during the LIA, especially since 330 cal yr BP, increased hurricane recurrence and higher precipitation variability would have delayed mangrove reestablishment. Charcoal influx was higher during the MCA, which was explained in terms of greater fire incidence due to enhanced biomass, and hence, fuel availability under wetter conditions [152].

Significant cultural events occurred during the last millennium, such as the crises of Maya civilization known as the Preclassic Abandonment (150–250 CE) and the Terminal Classic Collapse (750–950 CE). These events have been linked to extreme droughts as a consequence of the ITCZ migration [104] and have no definite imprints on the available mangrove records. Some suggestions based on chronological correlations exist, but straightforward evidence of the potential effects of these cultural shifts on mangroves is lacking. The same is true for European contact and its ensuing effects on indigenous populations and lifestyles, as well as on ecosystem disturbance patterns.

## 5. Conclusions and Prospects for Future Studies

Almost 60 Quaternary mangrove records have been identified in the Caribbean region, which have been useful to review the dynamics of these coastal communities and their natural and anthropogenic drivers of ecological change. The oldest records date back to ~130 kyr BP; no records have been found prior to the Late Pleistocene. A few records contain evidence from the Last Interglacial (MIS 5e or Eemian), and only one encompasses the Last Glaciation (Weichselian). The remaining 55 records (93%) correspond to the Middle and Late Holocene, as the Early Holocene is also absent.

During the Eemian, average temperatures and sea levels were higher than the present, and mangroves were in today’s continental areas, where some relictual patches still remain. No mangrove records have been found for the first part of the Last Glaciation (MIS 5d-a; ~115–70 cal kyr BP), when temperatures significantly decreased and sea levels were similar to or slightly below the present. The Caribbean mangrove record resumes in the coldest glacial phase (~70 cal ka BP onward), when sea levels were up to 120 m below the present. During those times, mangroves responded to the succession of Heinrich stadials and Dansgaard-Oeschger interstadials by alternating abundance declines and increases, respectively, always as minor components of coastal vegetation. Records corresponding to the Last Glacial Maximum (LGM: ~21 cal kyr BP), the Late Glacial (up to 11.7 cal yr BP), and the early Holocene (up to 8200 cal yr BP) are lacking, probably because the coasts were far from present ones and sedimentary mangrove records, if they exist, are now submerged under a water column between ~20 and 120 m deep. Holocene mangrove records begin at ~8000 cal yr BP, when climates were similar to today and sea levels were slightly lower. During those times, mangroves responded primarily to the sustained logarithmic-like eustatic rise characteristic of the Holocene.

Until 6000–5500 cal yr BP, natural drivers were the main external forcings of ecological dynamics, but since those dates, a new element, humans, came into action. Human societies entered the Caribbean coasts through Central America (13.5–12.5 cal yr BP) and colonized the easternmost continental Caribbean region by ~8 cal yr BP. The Greater Antilles were settled from Central America by 7–6 cal yr BP (Lithic/Archaic Age), and the Lesser Antilles were reached by humans between 4 (Archaic Age) and 2 cal yr BP (Ceramic Age). The first evidence of anthropogenic mangrove disturbance on the Caribbean coasts was found in Mesoamerica between 5600 and 5500 cal yr BP, when Preclassic Maya societies cleared mangrove forests using fire for maize and squash cultivation. This has been called the Mesoamerican Archaic Disturbance (MAD) event and is among the oldest records of agricultural practices in the region. In other Caribbean regions, human influence on mangroves was less important (except, perhaps, for a site in the Orinoco Delta) until the Late Holocene, when evidence of anthropogenic mangrove disturbance was widespread across the Caribbean region.

The Late Holocene began at 4200 cal yr BP with a regional millennial-scale climatic reversal, called here the Cariaco Holocene Instability (CHI) event, characterized by high precipitation variability and the occurrence of extreme droughts, probably due to the interplay between latitudinal ITCZ migrations and the intensification of ENSO activity. This event has been considered a major driver for mangrove reduction in many Caribbean areas, where drier climates could have favored salinity increases through enhanced evaporation and reduced freshwater input from rivers, thus creating unfavorable conditions for mangrove growth. The occurrence of potential climate-human amplification feedbacks has also been considered. The balance between sediment input from the continent and sea-level rise seems to have been crucial for mangrove dynamics. When this balance is biased toward continental terrigenous input, coastal progradation may offset eustatic rising and favor seaward migration of mangrove communities. Conversely, when sea-level rise is dominant, landward migration occurs. Several examples of this differential dynamics have been found during the Late Holocene as a manifestation of spatial heterogeneity across the Caribbean region.

During the last millennium, the most important drivers of mangrove change have been climate and human disturbance. The LIA, characterized by cold and dry climates, has negatively influenced mangrove development. Extreme meteorological events, such as hurricanes and destructive storms, have also played a significant role in mangrove reduction. An additional observation is that mangrove recovery after hurricane disturbance has been faster during wet climatic phases, such as the MCA, and more difficult under LIA dry conditions. Regarding human activities, mangrove deforestation for wood extraction and other practices such as fisheries, coconut plantations and rice crops have been among the better documented in paleoecological and historical records. The above-mentioned spatial heterogeneity has continued and has been attributed to differential local factors such as coastal geomorphology, fluvial dynamics and human disturbance, whereas the effects of climatic shifts have been of more regional reach. Interestingly, no paleoecological records of mangrove disturbance linked to relevant cultural events such as the Maya Preclassic and Classic demises (~200 CE and ~800 CE, respectively) or the European contact have been found.

To be complete, a review should be able to identify relevant knowledge gaps to be filled with future studies. In this case, the lack of mangrove records for the whole Quaternary is a major void that requires additional research effort. Finding a complete Pleistocene-Holocene record seems feasible only in marine records, ideally as part of the International Ocean Discovery Program (IODP; https://www.iodp.org/, accessed on 24 August 2022) or similar initiatives. Some of the Late Pleistocene pollen records discussed in this paper were obtained from marine corps retrieved in the Venezuelan Cariaco Basin, where older sequences encompassing the last 600 kyr BP were retrieved within the Ocean Drilling Program (ODP), the IODP precursor, and were studied for other paleoclimatic and paleoceanographic proxies [74,75]. It would be interesting to find out whether there is still enough material left in these cores to develop paleoecological studies on mangrove dynamics along the Pleistocene glacial-interglacial succession. In addition to this, the possibility of obtaining new full Pleistocene sequences in this basin is worth exploring. Obtaining a full Quaternary mangrove record for the Caribbean region is required to complete the sequence available to date that includes the Eocene origin and further Oligocene and Neogene evolution of these Neotropical communities [20,21,22], contributing to a thorough and integrated view of the ecological, biogeographical and evolutionary history of Caribbean mangroves. In addition to providing clues for the Quaternary history of Caribbean mangroves, long marine records could be useful for comparison with a unique continental record from central Colombia that encompasses the entire Pleistocene and has been intensively studied palynologically [199].

Obviously, the search for more Holocene mangrove records along present Caribbean coasts should continue, but the possibility of using the information gathered to date and reviewed in this paper to look for more focused targets is worth considering. For example, the potential for areas of known archaeological and historical interest to provide records encompassing the last millennia should be assessed with the aim of unravelling how relatively recent cultural changes have affected mangrove development. Special emphasis might be placed on critical phases for which this type of evidence is still lacking, as, for example, the crises of the Maya culture, the consequences of European contact, or the responses to the global warming of the last few centuries. This would help not only to understand how current mangrove communities have been shaped but also to inform conservation actions, especially in relation to the existence of potentially irreversible thresholds of climate change, sea-level rise and/or anthropogenic stress.

## Figures and Tables

**Table 2:** Phytosociological groups/subgroups defined for Neotropical mangrove formations [47]. *Aa*, *Acrostichum aureun*; *Ag*, *Avicennia germinans*; *Ce*, *Conocarpus erectus*; *Lr*, *Laguncularia racemosa*; *Pr*, *Pelliciera rhizophorae*; *Rm*, *Rhizophora mangle*.

Groups	Associations	Main Trees	Other Trees	Exclusive Species
A	Lag-AviP	*Ag*	*Lr*	*Sarcocornia pacifica*
	Lag-AviR	*Ag-Lr*	*Ce*	*-*
	Lon-LacC	*Lr*	*Ag-Ce*	*Lonchocarpus sericeus*, *Mimosa pigra*, *Phyllathus elsiae*
	Sta-LagC	*Lr*	*Ag*	*-*
	LagracR	*Lr*	*-*	*-*
B_1_	Acr-ConB	*Ce-Aa*	*Lr*	*Cladium mariscus*, *Acrostichum danaefolium*, *Eleocharis cellulosa*, *Fimbristylis spadicea*, *Ipomoea triloba*, *Schoenoplectus americanus*, *Helietta plaeana*, *Quadrella odoratissima*, *Acacia macracantha*
	Lon-ConC	*Ce*	*Rm-Lr-Ag*	*Lonchocarpus pycnophyllus*
	Spo-ConM	*Ce*	*-*	*Jacquinia keyensis*, *Pilosocereus royenii*, *Pithecelobium mucronatum*, *Tillandsia recurvata*
	Dis-ConR	*Ce*	*-*	*Paspalum distachyon*, *Chloris elara*
	Bat-ConM	*Ce*	*-*	*Evolvulus convolvuloides*, *Fimbristylis cimosa*, *Selenicereus grandiflorus*, *Broughtonia lindenii*, *Cienfuegosia yucatanensis*, *Suaeda linearis*, *Ambrosia hispida*
	Rac-ConM	*Ce*	*-*	*-*
	ConereR	*Ce*	*-*	*Lantana involucrata*, *Casasia clusiifolia*, *Ernodea littoralis*
	Con-CocR	*Ce*	*-*	*Thespecia populnea*
	Gra-ConC	*Ce*	*Lr-Ag*	*Pterocarpus acapulcensis*, *Phoradendron quadrangulare*
	Lag-ConP	*Ce*	*Lr*	*-*
B_2_	Lyc-ConR	*Ag-Ce*	*-*	*Chloris barbata*, *Cissus verticillata*, *Harrisia eriophora*, *Desmanthus virgatus*, *Lycium carolinianum*, *Opuntia dillenii*
	Sar-AviM	*Ag*	*Lr*	*-*
	Bat-AviB	*Ag*	*Ce*	*Sporobolus pyramidatus*, *Heliotrophium curassavicum*, *Iva cheiranthifolia*
	AvigerR	*Ag*	*-*	*Bastardia viscosa*
	Bat-AviP	*Ag*	*-*	*Monanthochloe littoralis*
	Avi-RhiR	*Ag-Rm*	*Ce*	*-*
C_1_	Pri-RhiC	*Rm*	*Aa*	*Prioria copaifera*, *Bactris guineensis*, *Curcumis melo*, *Inga alba*, *Bignonia hyacinthina*, *Crescentia cujete*, *Dalbergia beownei*, *Melothria pendula*
	Avi-RhiB	*Rm*	*-*	*Avicennia schaueriana*
	Ann-FicC	*Rm*	*-*	*Casearia aculeata*, *Erythrina fusca*, *Klarobelia anomala*
	RhimanC	*Rm*	*-*	*-*
C_2_	Pel-RhiC	*Rm-Lr-Pr*	*Ag*	*-*
	Rha-LagC	*Lr*	*Rm-Ag*	*Cyperus alternifolius*, *Echinochloa polystachia*, *Eleocharis interstincta*, *Heterostachys ritteriana*, *Eleocharis mutata*, *Ludwigia octovalvis*, *Persicaria acuminata*
	Mac-RhiC	*Rm*	*Lr-Ag*	*Nephrolepis multiflora*, *Dalbergia berteroi*, *Sabal causiarum*
	Dai-RhiB	*Rm*	*Lr*	*Bucida palustris*, *Hohenbergia penduliflora*, *Tillandsia usneoides*, *Sabal palmetto*, *Roystonea regia*, *Cissus trifoliata*, *Tillandsia fasciculata*, *Tabebuia angustata*
	Lag-RhiP	*Rm-Lr*	*-*	*Paullinia fuscescens*, *Blepharodon mucronatum*, *Albizia saman*, *Eclipta prostrata*, *Ficus bullenei*, *Gliricidia sepium*, *Maclura tinctoria*, *Mesechites trifidus*, *Solanum jamaicense*, *Terminalia catappa*, *Sacrostemma glaucum*, *Wedelia fruticosa*

## Data Availability

Data are provided in the manuscript.
